# Multi-cohort, multi-sequence harmonisation for cerebrovascular brain age

**DOI:** 10.1162/IMAG.a.964

**Published:** 2025-10-27

**Authors:** Mathijs B.J. Dijsselhof, Candace Moore, Saba Amiri, Mervin Tee, Saima Hilal, Christopher Chen, Bert-Jan H. van den Born, Wibeke Nordhøy, Ole A. Andreassen, Lars T. Westlye, Nishi Chaturvedi, Alun D. Hughes, David M. Cash, Jonathan M. Schott, Carole H. Sudre, Frederik Barkhof, Joost P.A. Kuijer, Francesca Biondo, James H. Cole, Henk J.M.M. Mutsaerts, Jan Petr

**Affiliations:** Department of Radiology and Nuclear Medicine, Amsterdam University Medical Centers, Vrije Universiteit, Amsterdam, The Netherlands; Amsterdam Neuroscience, Brain Imaging, Amsterdam, The Netherlands; Netherlands eScience Center, Amsterdam, The Netherlands; Saw Swee Hock School of Public Health, National University of Singapore and National University Health System, Singapore, Singapore; Memory Aging and Cognition Centre, National University Health System, Singapore, Singapore; Department of Pharmacology, Yong Loo Lin School of Medicine, National University of Singapore, Singapore, Singapore; Amsterdam UMC, University of Amsterdam, Department of Internal Medicine, Section Vascular Medicine, Amsterdam Cardiovascular Sciences, Amsterdam, The Netherlands; Amsterdam UMC, University of Amsterdam, Department of Public and Occupational Health, Amsterdam Public Health Research institute, Amsterdam, The Netherlands; Department of Physics and Computational Radiology, Division of Radiology and Nuclear Medicine, Oslo University Hospital, Oslo, Norway; Department of Psychology, University of Oslo, Oslo, Norway; Center for Precision Psychiatry, Division of Mental Health and Addiction, Oslo University Hospital, Oslo, Norway; KG Jebsen Centre for Neurodevelopmental Disorders, University of Oslo, Oslo, Norway; MRC Unit for Lifelong Health & Ageing, Institute of Cardiovascular Science, University College London, London, United Kingdom; Population Sciences and Experimental Medicine, Institute of Cardiovascular Science, University College London, London, United Kingdom; Dementia Research Centre, UCL Queen Square Institute of Neurology, London, United Kingdom; UK Dementia Research Institute at UCL, University College London, London, United Kingdom; Hawkes Institute, Department of Computer Science, University College London, London, United Kingdom; Department of Biomedical Computing, School of Biomedical Engineering & Imaging Sciences, King’s College London, London, United Kingdom; Queen Square Institute of Neurology, University College London, London, United Kingdom; Helmholtz-Zentrum Dresden-Rossendorf, Institute of Radiopharmaceutical Cancer Research, Dresden, Germany

**Keywords:** brain age, cerebrovascular ageing, cerebral blood flow, harmonisation, arterial spin labelling, machine learning

## Abstract

Higher brain-predicted age gaps (BAG), based on anatomical brain scans, have been associated with cognitive decline amongst elderly participants. Adding a cerebrovascular component, in the form of arterial spin labelling (ASL) perfusion MRI, can improve the BAG predictions and potentially increase sensitivity to cardiovascular health, a contributor to brain ageing and cognitive decline. ASL acquisition differences are likely to influence brain age estimations, and data harmonisation becomes indispensable for multi-cohort brain age studies including ASL. In this multi-cohort, multi-sequence study, we investigate harmonisation methods to improve the generalisability of cerebrovascular brain age. A multi-study dataset of 2608 participants was used, comprising structural T1-weighted (T1w), FLAIR, and ASL 3T MRI data. The single scanner training dataset consisted of 806 healthy participants, age 50 ± 17, 18–95 years. The testing datasets comprised four cohorts (n = 1802, age 67 ± 8, 37–90 years). Image features included grey and white matter (GM/WM) volumes (T1w), WM hyperintensity volumes and counts (FLAIR), and ASL cerebral blood flow (CBF) and its spatial coefficient of variation (sCoV). Feature harmonisation was performed using NeuroComBat, CovBat, NeuroHarmonize, OPNested ComBat, AutoComBat, and RELIEF. ASL-only and T1w+FLAIR+ASL brain age models were trained using ExtraTrees. Model performance was assessed through the mean absolute error (MAE) and mean BAG. ASL feature differences between cohorts decreased after harmonisation for all methods (p < 0.05), mostly for RELIEF. Negative associations between age and GM CBF (b = -0.37, R^2^ = 0.13, unharmonised) increased after harmonisation for all methods (b < -0.42, R^2^ > 0.12), but weakened for RELIEF (b = -0.28, R^2^ = 0.14), In the ASL-only model, MAE improved for all harmonisation methods from 11.1 ± 7.5 years to less than 8.8 ± 6.2 years (p < 0.001), while BAGs changed from 0.6 ± 13.4 years to less than -1.03 ± 7.92 years (p < 0.001). For T1w+FLAIR+ASL, MAE (5.9 ± 4.6 years, unharmonised) increased for all harmonisation methods non-significantly to above 6.0 ± 4.9 years (p > 0.42) and significantly for RELIEF (6.4 ± 5.2 years, p = 0.02), while BAGs non-significantly differed from -1.6 ± 7.3 years to between -1.3 ± 4.7 and -2.0 ± 8.0 years (p > 0.82). In general, the ASL-specific parameter harmonisation method AutoComBat performed nominally best. Harmonisation of ASL features improves feature consistency between studies and also improves brain age estimations when only ASL features are used. ASL-specific parameter harmonisation methods perform nominally better than basic mean and scale adjustment or latent-factor approaches, suggesting that ASL acquisition parameters should be considered when harmonising ASL data. Although multi-modal brain age estimations were improved less by ASL-only harmonisation, possibly due to weaker associations between age and ASL features compared with T1w features importance, studies investigating pathological ASL-feature distributions might still benefit from harmonisation. These findings advocate for ASL-parameter specific harmonisation to explore associations between cardiovascular risk factors, brain ageing, and cognitive decline using multi-cohort ASL and cerebrovascular brain age studies.

## 1 Introduction

Ageing is associated with the decline of physiological health and the development of pathology (Franke et al., 2020). The brain appearing older than normal for its chronological age is associated with increased risks of cognitive decline and mortality (Biondo et al., 2022; Cole et al., 2018). The brain-predicted age gap (BAG) is defined as the difference between the neuroimaging-derived predicted biological age and chronological age. BAG has shown value in predicting the risk of neurodegenerative pathology and psychiatric disorders, and determining and monitoring treatment strategies (Baecker et al., 2021). Commonly used neuroimaging-derived features to determine BAG are macroanatomical brain features such as GM and WM volume, and recent approaches have included multi-modality features to increase accuracy and sensitivity to functional and physiological brain ageing, and to predict the development of specific pathologies (Jirsaraie et al., 2023).

Cerebrovascular health markers are good extensions for BAG assessment as cerebrovascular pathology plays a role in many diseases ultimately leading to cognitive decline, such as vascular dementia (Pantoni, 2010) or Alzheimer’s Disease (AD) (Iadecola & Gottesman, 2019; Iturria-Medina et al., 2016). Additionally, it is essential to monitor the effects of cardiovascular health management in the cerebrovascular system (Dolui et al., 2022; Tryambake et al., 2013) due to its direct impact on cerebrovascular health (Williamson et al., 2018). Therefore, incorporating cerebrovascular health features into BAG may increase sensitivity to the risk of cognitive decline and mortality, and provide valuable insights into the impact of cardiovascular health (e.g., blood pressure change, coronary heart disease) and its (interventional) treatment on brain physiology and pathology.

An established method for non-invasively assessing cerebrovascular health is Arterial Spin Labeling (ASL) perfusion MRI (Alsop et al., 2015; Clement et al., 2022; Lindner et al., 2023). ASL-derived cerebral blood flow (CBF) has been shown to correlate with cognitive decline (Binnewijzend et al., 2013; van Dinther et al., 2024), amyloid-β and tau pathology (Falcon et al., 2024), and synaptic dysfunction (Falcon et al., 2024). The spatial coefficient of variation (sCoV) of CBF, as a measure of signal heterogeneity and a proxy of arterial transit time, is associated with ageing (Mutsaerts et al., 2017), atherosclerotic risk (Hafdi et al., 2022), and cognitive decline (Morgan et al., 2021). Improved BAG accuracy and classification of AD patients were shown by adding ASL features to commonly used T1w and FLAIR features, dubbed “cerebrovascular brain age”, in a single-cohort study (Dijsselhof et al., 2023; Rokicki et al., 2021). Although promising, these pre-trained single-cohort models do not generalise well to ASL datasets as a large variety of ASL implementations exist, with differences in acquisition hardware, labelling, and readout methods, and acquisition parameters (Grade et al., 2015). Furthermore, ASL acquisition parameters interact with the physiological state (Clement et al., 2018), resulting in additional differences between cohorts. All these factors influence the quantified perfusion values (Mutsaerts et al., 2015), introducing undesirable variability to the modelling of the relationship of age or disease with perfusion. One way to circumvent these issues is to train cerebrovascular brain age models using a mixture of datasets that cover the differences in ASL sequence parameters. Unfortunately, individual ASL studies tend to be limited in size, lack healthy controls, and cover a narrow age range (de Lange et al., 2022). Harmonisation is thus needed to reduce site- or sequence-related biases, commonly referred to as batch effects (Hu et al., 2023), and improve the crucial generalisability of cerebrovascular brain age estimations (Gaser et al., 2024).

In recent years, several harmonisation methods were developed that aim to minimise non-biological variance driven by aspects such as acquisition technique difference, while preserving biological and pathological associations and increasing power (Hu et al., 2023). The first methods correct the mean and variance of features across sites, such as NeuroComBat (Fortin et al., 2017), or include between-feature covariance estimates, such as in CovBat (A. A. Chen et al., 2022). Later methods attempted to address and retain complex associations between the harmonised features and biological covariates. NeuroHarmonize (Pomponio et al., 2020) models non-linear correlations with age, addressing possible non-linear correlations of age with arterial transit time (ATT) and CBF. These methods remain limited to a single batch effect estimation, while Complex associations between sites, scanners, and ASL acquisition parameters might need to be resolved through multiple harmonisation steps. OPNested ComBat with multiple ASL-specific batch variables might offer more flexibility to harmonise similar and distinctive ASL sequences (Horng et al., 2022). Furthermore, small differences in ASL acquisition parameters, such as the post-labelling delay (PLD), might result in similar batch effects, and AutoComBat mitigates sequence-dependent variations by clustering subjects into automatically identified batches by assessing several image acquisition parameters (Carré et al., 2022). Lastly, these approaches assume all batch effects are known, while this may not be the case, and identification of latent batch effects might further improve harmonisation. RELIEF incorporates prior batch effect knowledge and estimates latent batch effects (Zhang et al., 2023) to deal with unknown batch effects and mitigate ASL sequence parameter differences. Although some methods have been applied to structural BAG (Lombardi et al., 2020; Marzi et al., 2024; Pomponio et al., 2020), their impact on ASL-related issues with heterogeneity across sites and sequences is still unknown.

Here, we investigate harmonisation methods to improve the generalisability of cerebrovascular brain age in six cohorts differing in age range, and ASL acquisition types and parameters. Specifically, we investigate (1) the ability of harmonisation methods to reduce the between-cohort bias in ASL features, (2) the effect of different harmonisation methods on the accuracy of solely cerebrovascular, and combined structural and cerebrovascular brain age predictions, and (3) similarities in BAG before and after harmonisation.

## 2 Methods

### 2.1 MRI datasets

Training data were drawn from two cohorts scanned at the same scanner: the healthy controls of the StrokeMRI cohort, obtained at two time points, and the Thematically Organized Psychosis (TOP) cohort (Rokicki et al., 2021). Both studies were approved by the Regional Committee for Medical Research Ethics and the Norwegian Data Inspectorate. Testing data were drawn from several population-based cohorts: the Healthy Life in an Urban Setting (HELIUS) (Snijder et al., 2017); Southall And Brent Revisited (SABRE) (Jones et al., 2020); Epidemiology of Dementia In Singapore (EDIS) (Wong et al., 2020); and Insight 46 (a sub-study of the MRC NSHD; the British 1946 birth cohort) (Lane et al., 2017) studies. The HELIUS study was approved by the ethical review board of the Amsterdam University Medical Center. EDIS was approved by the National Healthcare Group-Specific Review Board and the Singapore Eye Research Institute. The National Research Ethics Service (NRES) Committee London granted ethical approval for SABRE (14/LO/0108) and Insight 46 (14/LO/1173). All participants provided written informed consent. Participants with mild cognitive impairment or dementia, as defined per cohort separately, or major brain pathology were excluded.

### 2.2 Imaging acquisition and processing

The study cohorts, MRI scanner and platform, and sequence parameters of the acquired structural T1-weighted (T1w) and T2-weighted (T2w) Fluid Attenuated Inversion Recovery (FLAIR), and ASL scans in each study are given in Table 1. Image processing was performed with ExploreASL version 1.11.0 (Mutsaerts et al., 2020) using Statistical Parametric Mapping 12 (SPM12), version r7219. Briefly, tissue segmentation of the structural T1w images into grey matter (GM), white matter (WM), and cerebrospinal fluid (CSF) was performed using the Computational Anatomy Toolbox 12 version r1615 (Gaser, 2009). WM hyperintensities (WMH) were segmented from FLAIR and used to fill WMH on T1w images ahead of segmentation using the lesion prediction algorithm of the Lesion Segmentation Toolbox version 2.0.15 (Schmidt et al., 2012). WMH volume and count (the number of spatially discrete clusters) were determined. Regions-of-interests (ROI) for GM were created as an intersection of the SPM12 GM, the anterior cerebral artery (ACA), the middle cerebral artery (MCA), and the posterior cerebral artery (PCA) (Tatu et al., 2012) ROIs with the individual CAT12 GM segmentations (partial volume > 0.7). ASL images were rigid-body registered to T1w images. The recommended single-compartment model was used to quantify CBF (Alsop et al., 2015). Partial-volume corrected mean CBF (Asllani et al., 2008) and the ASL sCoV—as the ratio of standard deviation divided by the mean (Mutsaerts et al., 2017)—were calculated in bilateral total GM and vascular territory ROIs. All images and ROIs were transformed to the Montreal Neurological Institute (MNI) standard space. CBF images were visually checked for typical artefacts (Alsop et al., 2015), such as inefficient labelling, excessive motion, or strong arterial transit time (ATT) artefacts, and if present were excluded from the study. Exclusion examples are shown in Supplementary Figure S1.

**Table 1.** Sequence parameters for the structural—T1w and T2w-FLAIR—and ASL images in the StrokeMRI, TOP, HELIUS, SABRE, EDIS, and Insight 46 cohorts.

Cohort	StrokeMRI/TOP	HELIUS	SABRE	EDIS	Insight 46
Vendor	GE MR750 3T	Philips Ingenia 3T	Philips Achieva 3T	Siemens Magnetom Trio Tim 3T	Siemens Biograph mMR 3T PET/MRI
**3D T1w structural**					
Sequence name	BRAVO	TFE	TFE	MPRAGE	MPRAGE
TR (ms)	8.16	7.01	6.80	2300	2000
TE (ms)	3.18	3.17	3.1	1.9	2.92
FA (deg)	12	9	8	9	8
Resolution (mm^3^)	1.00 x 1.00 x 1.00	1.00 x 1.00 x 1.00	1.00 x 1.00 x 1.00	1.00 x 1.00 x 1.00	1.10 x 1.10 x 1.10
**3D T2w-FLAIR**					
Sequence name	IR-CUBE	IR-VISTA		IR-SPACE	
TR (ms)	8000	4800	4800	9000	5000
TE (ms)	128	147	125	82	402
TI (ms)	2240	1650	1650	2500	1800
FA (deg)	Variable	Variable	Variable	Variable	Variable
Resolution (mm^3^)	1.00 x 1.00 x 1.20	1.10 x 1.10 x 1.12	1.00 x 1.00 x 1.00	1.00 x 1.00 x 3.00	1.10 x 1.10 x 1.10
**ASL**					
LD (ms)	1450	1800	1800	1500	1800
PLD (ms)	2025	1800	2000	1500	1800
TR (ms)	5025	4450	4615	4000	4000
TE (ms)	11.1	16.8	15	9.1	20.26
FA (deg)^a^	90/111	90/180	90/180	90/180	90/160
Nominal resolution (mm/pixels)	3.8 x 3.8 (512 points/8 arms)	3.00 x 3.17	3.75 x 3.75	3 x 3	3.75 x 3.75
Slice thickness (mm)	3	7	5	5	4
Background suppression	Yes	Yes	No	No	Yes
Labelling type	PCASL	PCASL	PCASL	PCASL	PCASL
Readout type	3D FSE Spiral	2D EPI	2D EPI	2D EPI	3D GRASE
Slice readout time (ms)	-	43.05	40.75	27.5	-
Averages	3	36	35	23	10
**M0**					
TR (ms)	2000	2000	9000	-^b^	2000
TE (ms)	11.1	13.1	15	-	18.8
Readout	3D FSE Spiral	2D EPI	2D EPI	-	3D GRASE
Slice readout time (ms)	-	18.85	22	-	-

M0 was not available in the EDIS study and the mean control image was used instead.

a Excitation and refocussing flip angles.

b The control images were used as calibration images in the CBF quantification.

ASL: arterial spin labelling; BRAVO: Brain Volume imaging; EPI: echo-planar imaging; FA: flip angle; FSE: fast spin-echo; GRASE: gradient and spin-echo; IR: inversion recovery; LD: labelling delay; MPRAGE: magnetisation prepared rapid gradient echo; M0: equilibrium magnetisation image; PCASL: pseudo-continuous ASL; PLD: post-labelling delay; SPGR: spoiled gradient; TE: echo time; TFE: turbo field echo; TI: inversion time; TR: repetition time; T1w: T1-weighted; T2w: T2-weighted.

### 2.3 Machine learning

To estimate unharmonised BAG, features from ASL-only, or including T1-weighted and FLAIR images together (T1w+FLAIR+ASL) were used. T1w features consisted of GM, WM, and CSF volumes, the ratio of GM to the intracranial volume (ICV), the ratio of both GM and WM to the ICV. Log-transformed FLAIR features consisted of the ratio of WMH volume divided by WM volume, and WMH count. ASL features consisted of both GM and vascular-territory-based CBF and log-transformed sCoV (Dijsselhof et al., 2023).

StrokeMRI and TOP were combined into a single training dataset, as both cohorts were obtained on the same 3T MR750 GE scanner with a 32-channel head coil, with the exact same scanner software release, sequences, and sequence parameters. The training was performed using the ExtraTrees algorithm and the full training dataset, and model performance was estimated through fivefold cross-validation stratified for age and sex in the same training dataset. The validation results were summarised across all folds and hereafter referred to as the Validation dataset. Testing was performed in the HELIUS, SABRE, EDIS, and Insight 46 cohorts to obtain the BAG and the mean absolute error (MAE; the mean absolute difference between the estimated and chronological brain age), and to determine the coefficient of determination (R^2^). Brain age estimations are commonly biased by the regression-to-the-mean effect (de Lange & Cole, 2020). The age bias was estimated across all cross-validation folds during training by scaling the predicted age of the testing cohorts by the slope and intercept from the regression of predicted age on chronological age in the validation cohort regressing the predicted age on chronological age (Supplementary Fig. S2) and subsequently applied to correct the predicted age in all testing cohorts (de Lange et al., 2022). Training, validation, and testing were performed separately for the ASL-only and T1w+FLAIR+ASL models.

### 2.4 ASL feature harmonisation

**Fig. 1.**
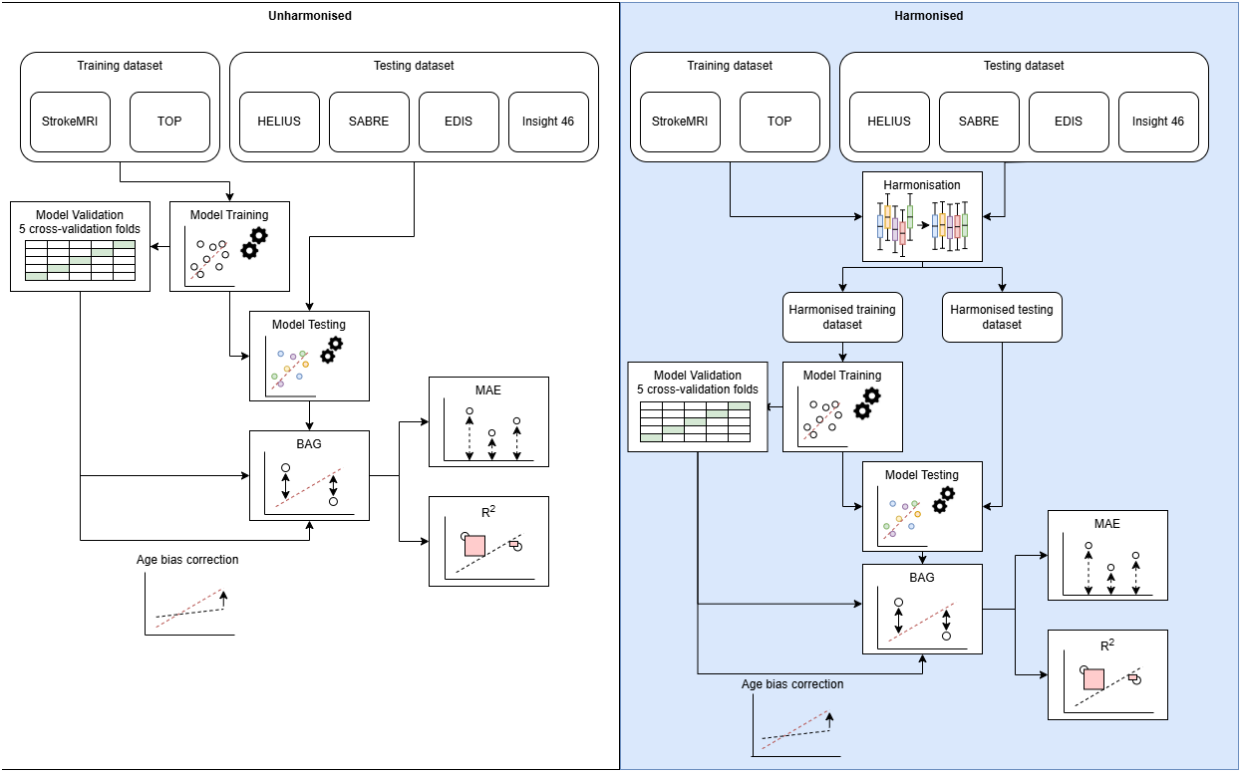
Brain age estimation model training and testing, and model performance evaluation, with (right) and without (left) data harmonisation. BAG: brain-predicted age gap; MAE: mean absolute error.

We tested the following feature-level statistical harmonisation methods mentioned by Hu et al. (2023): NeuroComBat (Fortin et al., 2017), CovBat (A. A. Chen et al., 2022), NeuroHarmonize (Pomponio et al., 2020), AutoComBat (Carré et al., 2022), OPNested ComBat (Horng et al., 2022), and RELIEF (Zhang et al., 2023). Each ASL feature was harmonised separately for all methods except CovBat, which incorporates harmonisation of feature covariances (A. A. Chen et al., 2022). NeuroComBat, CovBat, NeuroHarmonize, and RELIEF utilised cohort as a discrete batch parameter, while AutoComBat and OPNested ComBat utilised cohort and readout type as discrete, and PLD and LD as continuous batch parameters. The ASL features of all datasets (training and testing) were harmonised for each harmonisation method separately, using age and sex as covariates. After harmonisation, the training (using only the training dataset) and fivefold cross-validation of the training set were performed for every harmonisation method separately. Next, testing (in EDIS, HELIUS, Insight 46, and SABRE datasets) and the abovementioned age-bias corrections were repeated per harmonisation method with the corresponding harmonised datasets. This process was repeated for the ASL-only and T1w+FLAIR+ASL models separately. In total, the five datasets (training, EDIS, HELIUS, Insight 46, and SABRE) were harmonised using six methods and brain age was trained for two models (ASL-only or T1w+FLAIR+ASL), resulting in 60 different dataset–harmonisation–model combinations (Fig. 1).

### 2.5 Statistical analyses

#### 2.5.1 Demographics

Statistical analyses were conducted in R version 4.3.1 (R Core Team, 2023). Before harmonisation, all imaging data were tested for normal distribution using the Shapiro–Wilk test. The ratio of WMH volume to WM volume, WMH count, and all sCoV features were log-transformed because of their right-skewed distributions. To test whether T1w, FLAIR, and ASL features differed between all cohorts before harmonisation, analysis of covariance (ANCOVA) corrected for age and sex with Tukey post hoc tests corrected was performed.

#### 2.5.2 ASL feature harmonisation

To compare the overall effect of each harmonisation method, the means of ASL features across all cohorts were compared between harmonisation methods using ANCOVA corrected for age and sex with Tukey post hoc tests. To compare the effect of each harmonisation method on the difference of ASL features between cohorts, ANCOVA corrected for age and sex with Tukey post hoc tests for all cohort combinations (cohort-pairs) was performed separately for each ASL feature and harmonisation method. To investigate the association between age and change in each ASL feature between unharmonised and harmonisation methods, linear regressions were performed, including an interaction term for the harmonisation methods. Additionally, an interaction term between each ASL feature and cohort was used to investigate differences in the association between the training dataset and each testing dataset.

#### 2.5.3 Brain age

To assess the effect of harmonisation on the ASL-only model performance, differences of MAE in the validation dataset were compared between every harmonisation method and without harmonisation, using ANOVA with Tukey post hoc tests. Similar tests were performed to assess the performance in the testing dataset.

The following analyses were performed in the testing datasets only. To investigate whether harmonisation changed the explained variance within each model across all cohorts, the R^2^ was determined. The effect of each harmonisation method on the MAE difference between cohorts was investigated using ANOVA corrected for sex with Tukey post hoc tests for all cohort combinations (cohort-pairs). The same methods were used to assess the effect of each harmonisation method on the difference of BAG between unharmonised and harmonised data, or difference of BAG between cohorts for each harmonisation method, or unharmonised separately. These analyses have been performed for the models using ASL-only and T1w+FLAIR+ASL features separately.

### 2.6 Sensitivity analyses

To understand the effect of age bias correction, all brain age statistical analyses comparing unharmonised and harmonised data across all cohorts were repeated for BAG and MAE values not corrected for age bias.

### 2.7 Post hoc

The effect of ASL-feature harmonisation on the overall MAE and BAG in the T1w+FLAIR+ASL models might be limited due to the possible high importance of the structural features in the model. In this case, a post hoc analysis will be performed to investigate the underlying reasons. The importance of the structural features in determining the BAG was assessed using Shapley values (Rozemberczki et al., 2022). To explore this hypothesis, all T1w, FLAIR, and ASL features were harmonised per feature separately using NeuroComBat harmonisation, as the most commonly used harmonisation method (Hu et al., 2023), and the effect of this cross-modality harmonisation was investigated. T1w+FLAIR+ASL model-derived MAE and BAG will be compared between unharmonised and NeuroComBat harmonised features using t-tests, and the R^2^ calculated. Differences in MAE and BAG between cohorts for the NeuroComBat harmonised results will be compared using ANOVA with Tukey post hoc tests. In all statistics, p < 0.05 was defined as statistically significant.

## 3 Results

### 3.1 Demographics

**Fig. 2.**
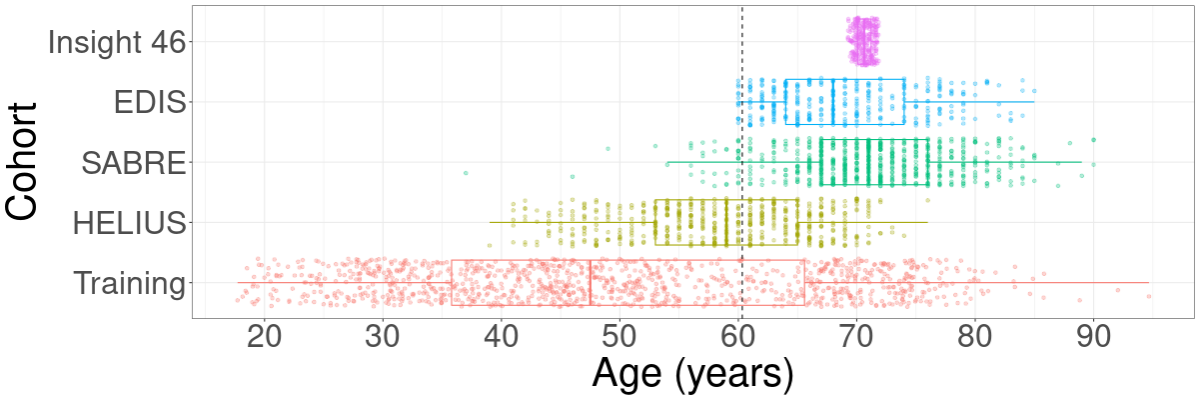
Age distribution per cohort. The training dataset consists of StrokeMRI and TOP combined. The dotted line represents the mean age of all cohorts combined.

After exclusion of participants without ASL scans (Training: 0; HELIUS: 15; SABRE: 22; EDIS: 413; Insight 46: 158) and dementia or major brain pathology (Training: 0; HELIUS: 0; SABRE: 0; EDIS: 7; Insight 46: 30), image quality was checked in participants with ASL scans, and 561 participants were excluded due to ASL artefacts (Training: 17; HELIUS: 40; SABRE: 64; EDIS: 191; Insight 46: 32; Supplementary Fig. S1), leaving a total of 2608 participants (Fig. 2; Table 2). All training dataset features differed between the population datasets (p < 0.05) before harmonisation, except for the WMH count between Training and HELIUS datasets (p = 0.80), ACA CBF between the Training and EDIS (p = 0.49), and PCA CBF between Training and Insight 46 (p = 0.15). Cohort differences are given in Supplementary Table S1.

**Table 2.** Demographics and imaging derivatives of the training and testing datasets.

Demographics	Training	HELIUS	SABRE	EDIS	Insight46
Participants (N)	806	531	642	346	282
Scans (N)	1094	531	642	346	282
Age [range] (years)	50.0 ± 17.0 [18 – 95]	58.6 ± 7.7 [39 – 76]	71.2 ± 6.6 [37 – 90]	68.9 ± 6.1 [60 – 65]	70.6 ± 0.7 [69 – 72]
Females (N, %)	605 (53%)	238 (45%)	281 (44%)	228 (66%)	149 (53%)
**Features**					
GM (L)	0.66 ± 0.07	0.57 ± 0.06	0.53 ± 0.05	0.53 ± 0.05	0.59 ± 0.05
WM (L)	0.51 ± 0.06	0.49 ± 0.07	0.45 ± 0.06	0.43 ± 0.06	0.49 ± 0.06
CSF (L)	0.35 ± 0.09	0.36 ± 0.09	0.36 ± 0.09	0.36 ± 0.08	0.38 ± 0.07
GM/ICV (ratio)	0.44 ± 0.04	0.41 ± 0.03	0.40 ± 0.03	0.40 ± 0.03	0.41 ± 0.02
(GM+WM)/ICV (ratio)	0.77 ± 0.05	0.75 ± 0.04	0.73 ± 0.05	0.73 ± 0.05	0.74 ± 0.03
WMH vol (mL)	2.95 (1.69, 5.66)	2.32 (1.43, 4.81)	3.42 (1.32, 8.16)	4.66 (2.46, 9.45)	2.67 (1.41, 4.47)
WMH/WM (ratio)	0.01 (0.00, 0.01)	0.00 (0.00, 0.01)	0.01 (0.00, 0.02)	0.01 (0.01, 0.02)	0.01 (0.00, 0.01)
WMH count (N)	3.14 (2.94, 3.33)	3.18 (2.94, 3.40)	3.14 (2.77, 3.47)	3.04 (2.83, 3.33)	3.00 (2.77, 3.30)
GM CBF (mL/100g/min)	82.41 ± 13.34	77.07 ± 12.73	58.98 ± 13.10	83.15 ± 18.56	77.42 ± 18.70
ACA CBF (mL/100g/min)	73.74 ± 12.07	68.03 ± 11.30	50.38 ± 12.32	81.78 ± 17.39	71.45 ± 17.84
MCA CBF (mL/100g/min)	58.15 ± 10.64	54.27 ± 9.84	43.63 ± 12.96	62.59 ± 14.88	56.76 ± 15.36
PCA CBF (mL/100g/min)	66.20 ± 11.29	61.27 ± 9.88	47.76 ± 11.47	72.33 ± 15.25	61.16 ± 14.79
GM sCoV (log(μ/σ))	-1.69 (-1.74, -1.62)	-1.09 (-1.16, -1.01)	-0.89 (-0.96, -0.77)	-0.67 (-0.80, -0.52)	-0.95 (-1.10, -0.76)
ACA sCoV (log(μ/σ))	-1.54 (-1.59, -1.48)	-0.99 (-1.05, -0.91)	-0.77 (-0.85, -0.65)	-0.57 (-0.70, -0.43)	-1.05 (-1.19, -0.88)
MCA sCoV (log(μ/σ))	-1.58 (-1.65, -1.49)	-0.98 (-1.08, -0.85)	-0.85 (-0.98, -0.70)	-0.45 (-0.58, -0.27)	-0.89 (-1.06, -0.73)
PCA sCoV (log(μ/σ))	-1.50 (-1.55, -1.43)	-0.96 (-1.03, -0.86)	-0.80 (-0.88, -0.69)	-0.52 (-0.66, -0.41)	-0.88 (-1.01, -0.72)

Unless stated otherwise, data are mean ± standard deviations or median (interquartile range).

WMH/WM, WMH count, and all sCoV features have been log-transformed. ACA: anterior cerebral artery; CBF: cerebral blood flow; CSF: cerebrospinal fluid; GM: grey matter; ICV: intracranial volume; MCA: middle cerebral artery; PCA: posterior cerebral artery; sCoV: spatial coefficient of variation; WM: white matter; WMH: white matter hyperintensities.

### 3.2 Feature harmonisation

For all cohorts combined (i.e., the full testing dataset), no differences in mean CBF and sCoV values (p > 0.05) were found between each harmonisation method and without harmonisation, however, the distribution was reduced after harmonisation (Supplementary Table S2A).

**Fig. 3.**
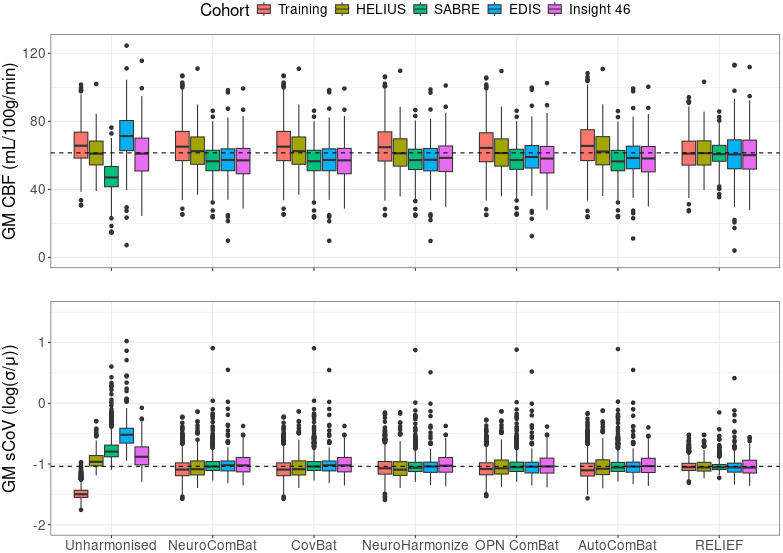
Boxplots of GM CBF and sCoV per cohort for every harmonisation technique. The box describes the second and third quartile range with the median. The dashed line represents the average unharmonised CBF value of all cohorts combined. CBF: cerebral blood flow; GM: grey matter; sCoV: spatial coefficient of variation.

GM CBF was different between 9 out of 10 cohort-pairs (p < 0.001) before harmonisation. The difference between cohorts in GM CBF decreased after harmonisation with seven cohort-pairs being different for NeuroCombat, CovBat, NeuroHarmonize, and AutoComBat (p < 0.001); six cohort-pairs for OPNested ComBat (p < 0.001), and no cohort-pairs for RELIEF (p > 0.99) (Fig. 3). The same results applied to ACA, MCA, and PCA CBF, with the exceptions of ACA CBF being different between eight cohort-pairs (p < 0.001) before harmonisation; six cohort-pairs for NeuroHarmonize (p < 0.01), and PCA CBF being different five cohort-pairs for OPNested ComBat (p < 0.01). All cohort and harmonisation-specific CBF values are given in the Supplementary Table S2B.

GM sCoV was different between all cohorts (p < 0.001) before harmonisation. The difference between cohorts in GM sCoV decreased after harmonisation (Supplementary Table S2A): with six cohort-pairs being different NeuroComBat and CovBat (p < 0.01); four cohort-pairs for NeuroHarmonize, OPNested ComBat, and AutoComBat (p < 0.03); and no cohort-pairs for RELIEF (p > 0.99) (Fig. 3). The same results applied to ACA, MCA, and PCA sCoV, with the exceptions of ACA sCoV being different between five cohort-pairs for NeuroComBat and CovBat (p < 0.04); two cohort-pairs for NeuroHarmonize, AutoComBat, and OPNested ComBat (p < 0.04); MCA sCoV being different between three cohort-pairs for NeuroComBat and CovBat (p < 0.05); one cohort-pair for OPNested ComBat (p < 0.02), and between no cohorts for NeuroHarmonize and AutoComBat (p > 0.16). Lastly, PCA sCoV was different between seven cohort-pairs for NeuroComBat and CovBat (p < 0.05); five cohort-pairs for OPNested ComBat (p < 0.02); and six cohort-pairs for AutoComBat (p < 0.04). All cohort and harmonisation-specific sCoV values are given in the Supplementary Table S2B.

**Fig. 4.**
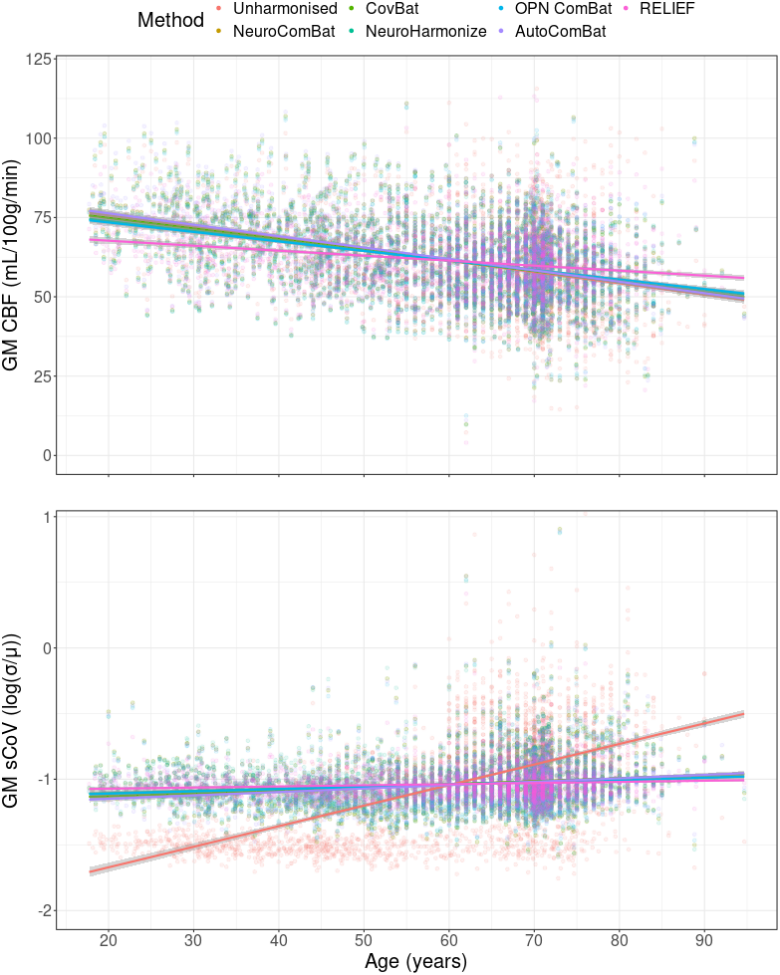
The associations of age with CBF and log-transformed sCoV in GM within the full testing dataset (n = 1801). CBF: cerebral blood flow; GM: grey matter; sCoV: spatial coefficient of variation.

Associations between age and GM CBF changed before and after harmonisations (Fig. 4A; Supplementary Table S3A). Age was associated with GM CBF (b = -0.37, CI between -0.41 and -0.34, R2 = 0.13, p < 0.001) before harmonisation, and the association increased for NeuroComBat, CovBat, NeuroHarmonize (b between -0.42 and -0.45, CI between -0.49 – and -0.39, R2 between 0.12 and 0.15, p < 0.03) and even more for AutoComBat (b = -0.47, CI between -0.51 and -0.44, R2 = 0.17, p = 0.001), decreased for RELIEF (b = -0.28, CI between -0.33 and -0.23, R2 = 0.14, p < 0.003), but did not change for OPNested Combat (b = -0.42, CI = -0.46, -0.38, p = 0.05). Age was similarly associated (p < 0.001) with ACA and MCA CBF (b = -0.31, CI between -0.34 and -0.28, R2 = 0.13), and PCA CBF (b = -0.29, CI between -0.33 and -0.25, R2 = 0.07) before harmonisation. For ACA CBF, the association with age decreased for NeuroCombat, CovBat and NeuroHarmonize, OPNested ComBat, and RELIEF (b between -0.17 and -0.30, CI between -0.32 and -0.13, R2 between 0.02 and 0.09), while increasing for AutoComBat (b = -0.32, CI between -0.36 and -0.29, R2 = 0.10), although RELIEF only significantly (p < 0.001) changed. Similar behaviour of the harmonisation methods to GM CBF was observed for MCA, although OPNested Combat also showed a significant decrease (p = 0.01), while for PCA CBF only NeuroCombat, CovBat, AutoComBat, and RELIEF showed similar significant change (p < 0.05). The associations between age and the CBF features differed (p < 0.001) between the training and testing datasets for all harmonisation methods.

Associations between age and sCoV values also changed before and after harmonisations (Fig. 4B; Supplementary Table S3B). Age was associated with GM sCoV (b = 20.53, CI between 19.44 and 21.61, R2 = 0.32, p < 0.001) before harmonisation, and the association decreased for all methods (b between 10.8 and 16.0, CI between 8.60 and 18.76, R2 between 0.01 and 0.04, p < 0.01) with AutoComBat retaining the strongest association (b = 16.0, CI between 13.17 and 18.76, R2 = 0.04, p = 0.003). Age was similarly associated (p < 0.001) with ACA (b = 18.8, CI between 17.75 and 19.74, R2 = 0.32), MCA (b = 18.9, CI between 17.84 and 19.98, R2 = 0.29), and PCA sCoV (b = 18.0, CI between 17.06 and 18.98, R2 = 0.32) before harmonisation. Similar behaviour of the harmonisation methods to GM sCoV was observed for ACA, MCA, and PCA sCoV, except NeuroComBat retained the strongest association with ACA sCoV (b = 8.7, CI between 6.03 and 11.46, R2 = 0.01, p < 0.001) and RELIEF with MCA sCoV (b = 5.7, CI between 0.28 and 11.19, R2 = 0.01, p < 0.001). All harmonisation methods changed compared with unharmonised associations of ACA, MCA, and PCA sCoV with age, except for PCA sCoV, which did not show significant change for AutoComBat (p = 0.53) and RELIEF (p = 0.11). The associations between age and GM sCoV differed between the training, EDIS, and Insight 46 for NeuroHarmonize (p < 0.05), and between all datasets for AutoComBat and RELIEF (p < 0.01). The associations of age with ACA differed the least between cohorts, followed by MCA and PCA after harmonisation (data not shown).

### 3.3 Brain age

#### 3.3.1 ASL-only model

**Fig. 5.**
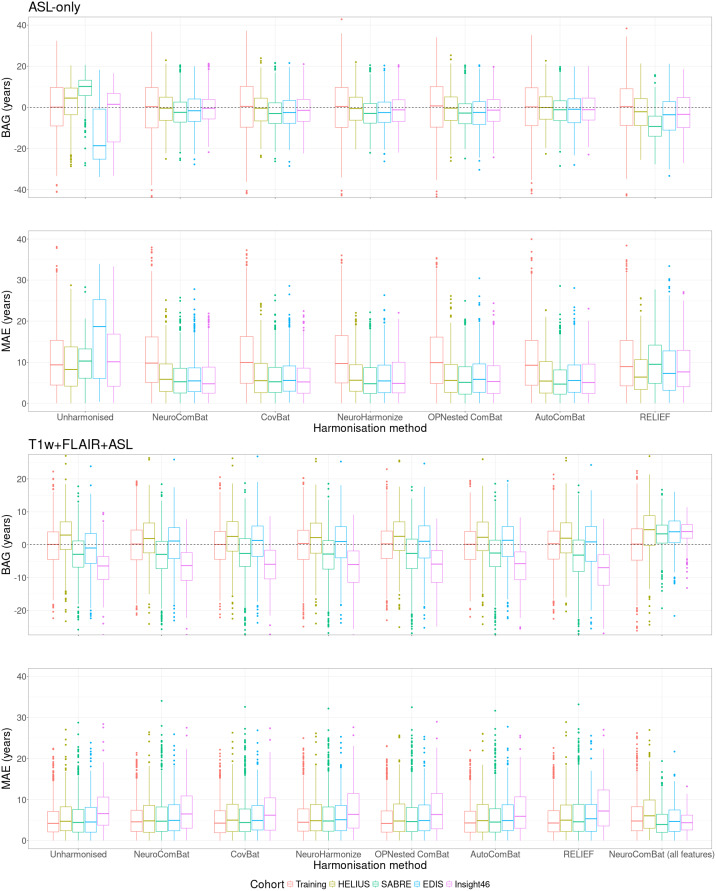
Boxplots of BAG and MAE of all cohorts per harmonisation method, obtained using ASL-only or T1w+FLAIR+ASL features to predict brain age. Harmonisation was performed on ASL features only. Additional results of harmonisation of all features using NeuroComBat have been included under “NeuroComBat (all features).” Note that the validation set was not corrected for age bias. ASL: arterial spin labelling; BAG: brain-predicted age gap; MAE: mean absolute error.

Using only ASL features (CBF and sCoV), the MAE (Table 3A; Fig. 5A) of unharmonised data was 10.65 ± 7.50 years in the validation set, and 11.08 ± 7.47 years in the testing set. In the validation dataset, the MAE did not differ statistically after harmonisation for all methods (p > 0.37, compared with unharmonised data). In the testing dataset, MAE differed statistically after harmonisation for all methods (p < 0.001, compared with unharmonised data), with 6.39 ± 4.87 years for NeuroComBat, 6.42 ± 4.87 years for CovBat, 6.32 ± 4.78 years for NeuroHarmonize, 6.54 ± 5.14 years for OPNested ComBat, 6.31 ± 4.89 years for AutoCombat, and 8.78 ± 6.15 years for RELIEF. RELIEF harmonisation differed statistically (p < 0.001) from all other methods, and all the other methods did not differ amongst themselves (p < 0.87, Supplementary Table S4A).

**Table 3.** BAG and MAE per harmonisation method for validation and testing sets, obtained using ASL-only or T1w+FLAIR+ASL features to predict brain age.

ASL-only	BAG (μ ± σ)	MAE (μ ± σ)	R^2^
**Validation dataset**			
Unharmonised	-0.23 ± 13.03	10.65 ± 7.50	0.40
NeuroComBat	-0.43 ± 13.88	11.32 ± 8.04	0.31
CovBat	-0.22 ± 13.74	11.19 ± 7.96	0.33
NeuroHarmonize	-0.24 ± 13.90	11.37 ± 8.00	0.31
OPNested ComBat	-0.12 ± 13.62	11.15 ± 7.81	0.34
AutoComBat	-0.18 ± 13.06	10.62 ± 7.61	0.39
RELIEF	-0.22 ± 12.91	10.42 ± 7.62	0.41
**Testing dataset**			
Unharmonised	0.61 ± 13.35	11.08 ± 7.47	0.23
NeuroComBat	-1.52 ± 7.89	6.39 ± 4.87	0.48
CovBat	-1.87 ± 7.84	6.42 ± 4.87	0.48
NeuroHarmonize	-1.76 ± 7.73	6.32 ± 4.78	0.47
OPNested ComBat	-1.94 ± 8.09	6.54 ± 5.14	0.44
AutoComBat	-1.03 ± 7.92	6.31 ± 4.89	0.47
RELIEF	-5.12 ± 9.42	8.78 ± 6.15	0.33

T1w+FLAIR+ASL	BAG(μ ± σ)	MAE(μ ± σ)	R^2^
**Validation dataset**			
Unharmonised	-0.07 ± 6.47	5.11 ± 3.98	0.85
NeuroComBat	0.11 ± 6.69	5.33 ± 4.03	0.84
CovBat	-0.08 ± 6.52	5.13 ± 4.01	0.85
NeuroHarmonize	0.11 ± 6.78	5.39 ± 4.11	0.84
OPNested ComBat	-0.06 ± 6.56	5.15 ± 4.06	0.85
AutoComBat	-0.05 ± 6.5	5.12 ± 4.00	0.85
RELIEF	-0.05 ± 6.61	5.21 ± 4.06	0.85
NeuroComBat (all features)	0.00 ± 7.30	5.77 ± 4.47	0.81
**Testing dataset**			
Unharmonised	-1.63 ± 7.28	5.88 ± 4.60	0.43
NeuroComBat	-1.75 ± 7.75	6.18 ± 4.99	0.43
CovBat	-1.30 ± 7.67	6.04 ± 4.91	0.43
NeuroHarmonize	-1.70 ± 7.78	6.21 ± 4.98	0.43
OPNested ComBat	-1.50 ± 7.76	6.15 ± 4.97	0.42
AutoComBat	-1.36 ± 7.64	6.04 ± 4.88	0.43
RELIEF	-2.00 ± 8.01	6.42 ± 5.17	0.41
NeuroComBat (all features)	3.56 ± 5.51	5.31 ± 3.85	0.63

Additional results of harmonisation of all features using NeuroComBat have been included under “NeuroComBat (all features).” Note that the validation set was not corrected for age bias.

ASL: arterial spin labelling; BAG: brain-predicted age gap; FLAIR: fluid attenuated inversion recovery; MAE: mean absolute error; T1w: T1-weighted.

In the testing dataset before harmonisation, the MAE of five out of six cohort-pairs was statistically different (p < 0.01, data not shown). After harmonisation, the MAE of no cohort-pairs was statistically different for NeuroComBat, CovBat, NeuroHarmonize, and OPNested ComBat (p > 0.13, data not shown), two cohort-pairs for AutoComBat (p < 0.03, data not shown), and five cohort-pairs for RELIEF (p = 0.99, data not shown). In the validation set, R^2^ did not change for RELIEF, however, decreased nominally for all other harmonisation methods compared with unharmonised data. In the testing set, R^2^ improved nominally for all harmonisation methods (Table 3A).

The unharmonised BAGs were -0.23 ± 13.03 and 0.61 ± 13.35 years for the validation and testing sets, respectively (Table 3A; Fig. 5A). In the validation dataset, BAGs did not differ statistically after harmonisation for all methods (p > 0.99, compared with unharmonised data). In the testing dataset, BAGs differed statistically after harmonisation for all methods (p < 0.001, compared with unharmonised data), with -1.52 ± 7.89 years for NeuroComBat, -1.87 ± 7.84 years for CovBat, -1.76 ± 7.73 years for NeuroHarmonize, -1.94 ± 8.09 years for OPNested ComBat, -1.03 ± 7.92 years for AutoComBat, and -5.12 ± 9.42 years for RELIEF. No harmonisation methods differed, except for RELIEF, which differed statistically from all other methods (p < 0.001) and AutoComBat, which differed from OPNested ComBat (p = 0.04, Supplementary Table S4A).

In the testing dataset before harmonisation, BAGs of all cohort-pairs were different (p < 0.001, data not shown). After harmonisation, BAGs of two cohort-pairs differed for NeuroComBat and CovBat (p < 0.05, data not shown), three cohort-pairs for NeuroHarmonize (p < 0.01, data not shown), and four cohort-pairs for OPNested ComBat (p < 0.03, data not shown) and RELIEF (p < 0.01, data not shown). No cohort-pairs were different for AutoComBat (p > 0.06, data not shown). Cohort-specific BAG and MAE distributions per harmonisation method are reported in Supplementary Table S5A.

#### 3.3.2 T1+ASL+FLAIR model

Using all features (T1w, FLAIR, CBF, and sCoV), the MAE (Table 3B; Fig. 5B) of unharmonised data was 5.11 ± 3.98 years in the validation set, and 5.88 ± 4.60 years in the testing set. In the validation dataset, the MAE did not differ statistically after harmonisation for all methods (p > 0.71, compared with unharmonised data). In the testing dataset, the MAE was not statistically different (p > 0.37) with 6.18 ± 4.99 years for NeuroComBat, 6.04 ± 4.91 years for CovBat, 6.21 ± 4.98 years for NeuroHarmonize, 6.15 ± 4.97 years for OPNested ComBat, 6.04 ± 4.88 years for AutoComBat, and increased to 6.42 ± 5.17 years for RELIEF (p = 0.02) compared with the unharmonised data. No harmonisation methods were statistically different (p > 0.23, Supplementary Table S4B). In the testing dataset before harmonisation, the MAE of three cohort-pairs was different (p < 0.01, data not shown), and remained different after harmonisation (p < 0.03, data not shown). In the validation set, R^2^ did not differ for all methods except for OPNested ComBat and RELIEF, where it nominally decreased, compared with unharmonised data (Table 3B). Similar results were obtained in the testing set.

The unharmonised BAGs (Table 3B; Fig. 5B) were, respectively, -0.07 ± 6.47 years and -1.63 ± 7.28 years for the validation and testing sets. In the validation dataset, BAGs did not differ statistically after harmonisation for all methods (p > 0.99, compared with unharmonised data). In the testing dataset, BAGs did not differ statistically after harmonisation for all methods (p > 0.78, compared with unharmonised data), with -1.75 ± 7.75 years for NeuroComBat, -1.30 ± 7.67 years for CovBat, -1.70 ± 7.778 years for NeuroHarmonize, -1.50 ± 7.76 years for OPNested ComBat, -1.36 ± 7.64 years for AutoComBat, and -2.00 ± 8.01 years for RELIEF. No BAGs of any harmonisation methods were different (p > 0.16, Supplementary Table S4B).

In the testing dataset before harmonisation, BAGs of all cohort-pairs were different (p < 0.001, data not shown), and remained different after harmonisation (p < 0.01, data not shown). Cohort-specific BAG and MAE distributions per harmonisation method are reported in Supplementary Table S5B.

#### 3.3.3 Sensitivity

Without age-bias correction, in the unharmonised testing dataset of the ASL-only model, the MAE was 13.24 ± 12.28 years (R^2^ = 0.02) and the BAGs were -10.46 ± 14.72 years. After harmonisation, the MAE increased to above 14.66 ± 8.68 years for NeuroComBat, CovBat, NeuroHarmonize, OPNested ComBat, and RELIEF (p < 0.001), while AutoComBat did not differ (13.31 ± 8.17 years, p = 0.99, Supplementary Table S6A). The BAGs decreased to below 12.02 ± 9.96 years (p < 0.001) for all methods. The R^2^ increased to between 0.03 and 0.08 for all methods except for RELIEF, which remained at 0.02.

In the unharmonised testing dataset of the T1w+ASL+FLAIR model, the MAE was 7.4 ± 5.42 years (R^2^ = 0.30), and the BAGs were -4.7 ± 7.88 years. After harmonisation, the MAE and BAGs did not differ for any method (p > 0.61, Supplementary Table S6B). The R^2^ increased to between 0.31 and 0.32 for all methods except RELIEF, which remained at 0.30.

#### 3.3.4 Post hoc analyses

The three most important features in the T1w+FLAIR+ASL model were the ratio of GM with ICV, the ratio of GM and WM combined with ICV, and the ratio of WMH volume with WM volume (Supplementary Fig. S3). After harmonisation of all features (T1w, FLAIR, and ASL) using NeuroComBat, the MAE (Table 3B; Fig. 5B) increased to 5.77 ± 4.47 years in the validation set (p < 0.01, compared with unharmonised data). In the testing dataset, the MAE decreased to 5.31 ± 3.85 years (p < 0.01) and differed statistically between four out of six cohort-pairs (p < 0.03). The BAGs did not change in the validation dataset with 0.00 ± 7.30 years (p = 0.99, compared with unharmonised data). In the testing dataset, the BAGs increased to 3.56 ± 5.51 years (p < 0.001) and differed statistically for one out of six cohort-pairs (p < 0.01). R^2^ decreased in the validation set and increased in the testing set compared with unharmonised data (Table 3B).

## 4 Discussion

### 4.1 Summary of results

We have investigated six feature-harmonisation methods and their effect on the generalisability of cerebrovascular brain age across five datasets differing in age distribution and ASL acquisition parameters. This study has three main findings. First, all harmonisation methods decreased the difference in ASL features (CBF and sCoV). RELIEF reduced differences in ASL features between cohorts the most. However, it also reduced the association between age and ASL features while all other harmonisation methods retained or even strengthened these associations. Second, cerebrovascular brain age performance improved (lower MAE) in all testing datasets for the ASL-only model after harmonisation. However, this was not the case for the combined (T1w+FLAIR+ASL) cerebrovascular brain age model, while harmonising all features did improve the combined cerebrovascular brain age performance. Third, with respect to the unharmonised counterpart, BAGs decreased for all methods in the ASL-only model and did not change in the combined model after harmonisation of only ASL features, but increased in the combined model after harmonisation of all features.

### 4.2 Features

Although harmonisation methods reduced mean cohort differences and standard deviations in ASL features, RELIEF harmonisation consistently achieved lower cohort differences. One explanation could be that inherent age-related CBF and sCoV differences between the cohorts are removed instead of preserved in RELIEF as its latent-factor approach does not force its latent variations to be independent of covariates, even when covariates such as age and sex are provided (Zhang et al., 2023). It is known that CBF decreases with age (J. J. Chen et al., 2011), and this pattern remains with all harmonisation methods except RELIEF. Moreover, RELIEF reduced associations between age and ASL features, whereas OPNested ComBat and AutoComBat showed the strongest age associations. The likely explanation is that OPNested ComBat and AutoComBat additionally accounted for the ASL acquisition parameters differences between cohorts, which are known to strongly affect their CBF measurements (Mutsaerts et al., 2015). The PLD plays a critical role in CBF quantification accuracy, especially in older and cerebrovascularly diseased populations, to account for arterial transit time prolongation that otherwise would result in underestimated CBF (Alsop et al., 2015).

The highest harmonisation effect on CBF and sCoV was found in the SABRE and EDIS cohorts. Unlike the training and other testing datasets with 3D readout, CBF in these datasets was acquired using a 2D EPI readout. This aligns with previously observed CBF measurement differences between 2D and 3D sequences, even when acquired on the same scanner (Baas et al., 2021). Because we used large regions, we expect this to be due to PLD differences between acquired slices or background suppression efficiency (effect of head motion) and M0 estimation rather than the effective spatial resolution and geometric distortion differences between 2D and 3D ASL acquisitions (Mutsaerts et al., 2014). The EDIS dataset also had considerably shorter PLD, which, combined with the lack of background suppression, could result in more noise, perhaps explaining the large harmonisation effect. Interestingly, the HELIUS dataset, scanned with a 2D EPI readout including background suppression, was less affected by harmonisation, likely due to the fact that its age range and PLD were more similar to that of the training dataset. AutoComBat, a harmonisation method that utilises ASL parameters to estimate batch effects obtained nominally the best results. This method may be especially useful in older populations, in the presence of pathology, and in 2D ASL acquisitions where the effects of PLD on measured CBF due to increased arterial transit time tend to be stronger (Damestani et al., 2023).

### 4.3 Brain age

Consistent with previous studies, brain age estimations using ASL-only features showed relatively low performance (higher MAE) compared with models using T1w, FLAIR, and ASL features (Dijsselhof et al., 2023; Rokicki et al., 2021), which can be attributed to the high physiological variability of perfusion (Clement et al., 2018). In the ASL-only model, validation dataset performance was not different after harmonisation although its variance was increased. As the training dataset had more stringent criteria for selecting healthy participants than the other included datasets (Rokicki et al., 2021), patterns of general ageing from the testing datasets were possibly introduced to the training dataset through the joint harmonisation of all datasets, resulting in this larger variance of the validation dataset.

AutoCombat performed non-significantly better on average than the other mean and scale adjustment methods (NeuroComBat, CovBat, NeuroHarmonize). However, AutoComBat did nominally reduce the variation of individual features and improve model fit the most, which could be attributed to the strengthened associations between the ASL features and age. Conversely, RELIEF showed the worst performance, which could be attributed to its weakened associations between age and ASL features. These results are similar to those of ASL feature harmonisation, further demonstrating that large ROIs, such as the whole GM or vascular territories, are not strongly affected by the interaction of ASL parameters, age, and measured perfusion. However, these interactions could be more prominently altered in cohorts where CBF and sCoV are affected by pathology (Grade et al., 2015; Gyanwali et al., 2021).

Model performance using all features (T1w, FLAIR, and ASL) did not improve after harmonising ASL features for most methods. The exception was RELIEF, which showed worse performance in both the validation and testing datasets. This can be attributed to the lower importance of ASL than T1w features for estimating brain age, likely due to the high variability of CBF within and between individuals (Clement et al., 2018). However, as a potential earlier biomarker than the structural change detected on T1w-MRI (Grade et al., 2015), ASL-derived CBF as a feature in estimating cerebrovascular ageing might play a greater role in the context of pathology than in healthy ageing.

Harmonisation in the ASL-only model did not change brain age interpretability (i.e., direction of the BAG) for the validation dataset. However, the interpretability changed for the testing datasets with all harmonisation methods resulting in the average BAG being negative, or the brain appearing younger than its chronological age, with RELIEF showing the largest change. This result suggests that harmonisation provides a more truthful image of ageing that is otherwise obscured due to ASL sequence differences, as the Insight 46 cohort on average appeared to be more healthy than its contemporaries (Dijsselhof et al., 2025; James et al., 2018), and has been estimated to be younger than its chronological age in another brain age study (Wagen et al., 2022). This, however, is difficult to confirm, as no brain age studies have been performed in the other datasets used here.

BAG direction did not change in the model using T1w, FLAIR, and ASL after harmonisation, again possibly due to the importance of ASL features in estimating brain age. Expectedly, harmonisation does not remove the inherent regression-to-the-mean effect that creates an age bias in brain age estimation models (de Lange & Cole, 2020). Uncorrected brain age estimations did change brain age interpretability, resulting in large underestimation and worse model accuracy, which can be explained by the older average age of the testing compared with the training datasets. The use of age-bias corrections is debated (de Lange & Cole, 2020), and other age bias correction methods may inflate model accuracy (Butler et al., 2021). Therefore, coupled with the differences found in this study, it remains important to assess model performance with and without correction.

### 4.4 Post hoc analyses

Brain age accuracy in the testing data improved significantly compared with the unharmonised estimations when NeuroCombat was used to harmonise T1w, FLAIR, and ASL features. This additionally highlights the higher importance of structural features over ASL features in brain age estimations, possibly due to the large inter-subject variability in CBF (Clement et al., 2018). Compared with the harmonisation of only ASL features and the results of the ASL-only brain age estimations, harmonising all features did have less effect on T1w+FLAIR+ASL brain age estimations. This could be explained by smaller differences between T1w sequences in volumetric brain measurements (van Nederpelt et al., 2023) compared with differences between ASL sequences (Almeida et al., 2018), as measured by intraclass correlation coefficients. The effect of T1w normalisation on the brain age estimation thus clearly highlights the role of feature importance on brain age estimations.

NeuroComBat harmonisation of all features resulted in a positive shift of average BAG (brain appearing older than chronological age). The largest BAG change occurred in the Insight 46 dataset, of which a previous study using T1w volumetric features showed an opposite result with a younger appearing age on average (Wagen et al., 2022). In these cases, where harmonisation changes the BAG interpretation, it is imperative to consider other measures of health and the unharmonised BAG.

The current feature-based approach renders the brain age estimation less sensitive to image-related sequence differences such as susceptibility artefacts or the presence of vascular ASL signal (Alsop et al., 2015). In contrast, deep-learning-based brain age estimations might be more susceptible to these issues, and other methods of image level harmonisations using deep- or transfer-learning approaches (Da-Ano et al., 2021; Hu et al., 2023) might need to be applied before these advanced estimation methods can be used. Additionally, ASL-derived CBF could be altered severely with pathology (Grade et al., 2015), affecting feature distributions. Lastly, many variations in ASL sequences exist that differ in labelling technique, PLD timings, and readout approaches (van Osch et al., 2018), but only a few have been included in this study. Additionally, there is further variety in image processing pipelines (Paschoal et al., 2024), adding to the between-center variability. Therefore, future studies are encouraged to investigate the effect of harmonisation on association of CBF and sCoV in diseased populations, and validate the positive effect of image-level harmonisations in the presence of pathology across a wider range of ASL sequences and processing pipelines.

### 4.5 Limitations

This study has several limitations. First, a major limitation of this study is the lack of a ground truth in population average CBF values and cerebrovascular brain age estimates, to assess the effect of the harmonisation methods. CBF is dependent on many physiological and pathological factors (Clement et al., 2018), and controlling for all factors is not feasible. Furthermore, many methods exist to assess the effect of harmonisation on feature distributions (Hu et al., 2023), however, there is no consensus about a universally applicable method. Contributing to the difficulty of establishing a ground truth, the population differences amongst the testing datasets are another limitation, which makes it challenging to disentangle ASL sequence batch effects from population differences. For example, Insight 46 has a very narrow age range, which complicates harmonisation of datasets with larger age ranges. Although age-matched subsets might offer more insight into the usefulness and correctness of harmonisations, large and homogeneous ASL datasets are not easily available, making the studied scenario a realistic example. Additionally, ASL sequence parameters will affect ASL signal distributions, derived CBF, and the presence of (e.g., motion, arterial transit) artefacts. For example, the short PLD of EDIS resulted in high sCoV (Mutsaerts et al., 2017) and CBF, and a higher exclusion rate. High CBF and high sCoV correspond to macrovascular signal presence, in contrast to the preferred perfusion signal, which is a major limitation of ASL (Iutaka et al., 2023). Additionally, while artefact-based exclusions can be considered vital in image analysis in research and clinical settings, this may bias the included data towards healthier participants compared with the other cohorts. While harmonisation appeared to improve cerebrovascular brain age estimations by reducing the MAE, further work is needed to study the effect of exclusion bias on the accuracy of the cerebrovascular age model and the feasibility of applying it to very low-quality datasets. The perfect solution would be to utilise travelling participants, which has been shown to outperform NeuroComBat harmonisation of the whole cohort (Maikusa et al., 2021), however, such data are currently not publicly available in the utilised ASL datasets.

Second, this study has investigated only a selection of feature-level harmonisation methods, and omitted deep-learning-based harmonisations (Hu et al., 2023), as the former is relatively straightforward to understand and interpret the effect of harmonisation on the feature-based brain age estimation. Although deep-learning methods might harmonise better than feature-level methods, specifically as they might better address spatial variations between ASL sequences, it is more difficult to understand their effect on the harmonisation of ROI-specific CBF and sCoV features. Within these feature-level methods, despite not all publicly available harmonisation methods being tested, the selection covers all groups of methods described by Hu et al. (2023), assuming that performance will be similar across the group.

Furthermore, many harmonisation methods assume normally distributed features. To avoid non-normality issues in this study, several features (WMH and sCoV) have been log-transformed, however, this decreases the interpretability of the individual measures. In general, and in this study specifically, training datasets suffer from fewer outliers due to the inclusion of healthy participants. However, harmonisation might be less effective in non-normally distributed features and will adversely affect the distribution in the training datasets if testing datasets include patients. Furthermore, this could result in worse model performance, as seen in the validation results in this study, and this effect could be even more pronounced when harmonising patient data. Therefore, other harmonisation methods that are able to handle non-normal distributions should be studied, especially in the context of pathology. Lastly, the preservation of biological and pathological associations by the harmonisation methods was assessed by determining associations between the ASL features and age in this study. This allows for only a partial view into assessing the (in)ability of the harmonisation methods to disentangle batch and population effects, as shown by the difference of RELIEF compared with the other harmonisation methods in the associations between ASL features and age. Lastly, relatively large ROI-based ASL features (GM and vascular territories) were used to determine cerebrovascular brain age, whereas pathological CBF changes are known to occur in smaller regions, such as in AD (Austin et al., 2011; Graff et al., 2023). However, a previous study has shown that ASL-based brain age estimation methods that utilise larger ROIs are more accurate than utilising smaller ROIs in BAG estimation in healthy controls (Dijsselhof et al., 2023), possibly due to the low ASL signal to noise and resulting higher noise in smaller regions. This leads to the choice of larger regions, although we acknowledge that it remains to be studied how harmonisation effects smaller ROIs, and how both large and small ROI-based cerebrovascular brain age models behave in the context of pathology. Exploring the associations of CBF features and brain age estimations with other health parameters, such as blood pressure and cognitive scores, and perfusion modifiers will allow for a better understanding of whether datasets-specific associations are retained while improving compatibility across datasets.

## 5 Conclusions

In the largest ASL brain age study to date, consisting of several datasets acquired with different ASL sequences and population characteristics, we showed that ASL-derived CBF and sCoV can be harmonised using traditional feature-level harmonisation methods. AutoComBat achieved the highest comparability and model accuracy when considering that latent-factor approaches might remove biological associations. While adding T1w and FLAIR features lowered the effect of harmonisation on brain age estimation performance. The improvement in ASL-feature-only model clearly showed the added value of ASL data harmonisation in multi-cohort ASL analyses, allowing advanced models such as brain age estimations to explore the associations between ageing, cardiovascular risk factors, brain health, and cognitive decline.

## Supplementary Material

Supplementary Material

## Data Availability

No new data were acquired for this study, and all data have been requested through the respective data request procedures of each study as detailed in the referenced publications. ExploreASL code is freely available at https://github.com/ExploreASL/ExploreASL. Harmonisation and brain age estimation code are under development and are available on request at https://github.com/ExploreASL/cvasl and https://github.com/MDijsselhof/CerebrovascularBrainAge and will be released under an open-source license in the future.

## References

[IMAG.a.964-b1] Almeida, J. R. C., Greenberg, T., Lu, H., Chase, H. W., Fournier, J. C., Cooper, C. M., Deckersbach, T., Adams, P., Carmody, T., Fava, M., Kurian, B., McGrath, P. J., McInnis, M. G., Oquendo, M. A., Parsey, R., Weissman, M., Trivedi, M., & Phillips, M. L. (2018). Test-retest reliability of cerebral blood flow in healthy individuals using arterial spin labeling: Findings from the EMBARC study. Magnetic Resonance Imaging, 45, 26–33. 10.1016/j.mri.2017.09.004 28888770 PMC5743013

[IMAG.a.964-b2] Alsop, D. C., Detre, J. A., Golay, X., Günther, M., Hendrikse, J., Hernandez-Garcia, L., Lu, H., MacIntosh, B. J., Parkes, L. M., Smits, M., van Osch, M. J. P., Wang, D. J. J., Wong, E. C., & Zaharchuk, G. (2015). Recommended implementation of arterial spin-labeled perfusion MRI for clinical applications: A consensus of the ISMRM perfusion study group and the European consortium for ASL in dementia. Magnetic Resonance in Medicine: Official Journal of the Society of Magnetic Resonance in Medicine / Society of Magnetic Resonance in Medicine, 73(1), 102–116. 10.1002/mrm.25607 PMC4190138 24715426

[IMAG.a.964-b3] Asllani, I., Borogovac, A., & Brown, T. R. (2008). Regression algorithm correcting for partial volume effects in arterial spin labeling MRI. Magnetic Resonance in Medicine, 60(6), 1362–1371. 10.1002/mrm.21670 18828149

[IMAG.a.964-b4] Austin, B. P., Nair, V. A., Meier, T. B., Xu, G., Rowley, H. A., Carlsson, C. M., Johnson, S. C., & Prabhakaran, V. (2011). Effects of hypoperfusion in Alzheimer’s disease. Journal of Alzheimer’s Disease: JAD, 26(Suppl. 3), 123–133. 10.3233/978-1-60750-793-2-253 21971457 PMC3303148

[IMAG.a.964-b5] Baas, K. P. A., Petr, J., Kuijer, J. P. A., Nederveen, A. J., Mutsaerts, H. J. M. M., & van de Ven, K. C. C. (2021). Effects of acquisition parameter modifications and field strength on the reproducibility of brain perfusion measurements using arterial spin-labeling. AJNR. American Journal of Neuroradiology, 42(1), 109–115. 10.3174/ajnr.a6856 33184068 PMC7814799

[IMAG.a.964-b6] Baecker, L., Garcia-Dias, R., Vieira, S., Scarpazza, C., & Mechelli, A. (2021). Machine learning for brain age prediction: Introduction to methods and clinical applications. EBioMedicine, 72, 103600. 10.1016/j.ebiom.2021.103600 34614461 PMC8498228

[IMAG.a.964-b7] Binnewijzend, M. A. A., Kuijer, J. P. A., Benedictus, M. R., van der Flier, W. M., Wink, A. M., Wattjes, M. P., van Berckel, B. N. M., Scheltens, P., & Barkhof, F. (2013). Cerebral blood flow measured with 3D pseudocontinuous arterial spin-labeling MR imaging in Alzheimer disease and mild cognitive impairment: A marker for disease severity. Radiology, 267(1), 221–230. 10.1148/radiol.12120928 23238159

[IMAG.a.964-b8] Biondo, F., Jewell, A., Pritchard, M., Aarsland, D., Steves, C. J., Mueller, C., & Cole, J. H. (2022). Brain-age is associated with progression to dementia in memory clinic patients. NeuroImage. Clinical, 36, 103175. 10.1016/j.nicl.2022.103175 36087560 PMC9467894

[IMAG.a.964-b9] Butler, E. R., Chen, A., Ramadan, R., Le, T. T., Ruparel, K., Moore, T. M., Satterthwaite, T. D., Zhang, F., Shou, H., Gur, R. C., Nichols, T. E., & Shinohara, R. T. (2021). Pitfalls in brain age analyses. Human Brain Mapping, 42(13), 4092–4101. 10.1002/hbm.25533 34190372 PMC8357007

[IMAG.a.964-b10] Carré, A., Battistella, E., Niyoteka, S., Sun, R., Deutsch, E., & Robert, C. (2022). AutoComBat: A generic method for harmonizing MRI-based radiomic features. Scientific Reports, 12(1), 12762. 10.1038/s41598-022-16609-1 35882891 PMC9325761

[IMAG.a.964-b11] Chen, A. A., Beer, J. C., Tustison, N. J., Cook, P. A., Shinohara, R. T., Shou, H., & Alzheimer’s Disease Neuroimaging Initiative. (2022). Mitigating site effects in covariance for machine learning in neuroimaging data. Human Brain Mapping, 43(4), 1179–1195. 10.1002/hbm.25688 34904312 PMC8837590

[IMAG.a.964-b12] Chen, J. J., Rosas, H. D., & Salat, D. H. (2011). Age-associated reductions in cerebral blood flow are independent from regional atrophy. NeuroImage, 55(2), 468–478. 10.1016/j.neuroimage.2010.12.032 21167947 PMC3435846

[IMAG.a.964-b13] Clement, P., Mutsaerts, H.-J., Václavu˚ , L., Ghariq, E., Pizzini, F. B., Smits, M., Acou, M., Jovicich, J., Vanninen, R., Kononen, M., Wiest, R., Rostrup, E., Bastos-Leite, A. J., Larsson, E.-M., & Achten, E. (2018). Variability of physiological brain perfusion in healthy subjects—A systematic review of modifiers. Considerations for multi-center ASL studies. Journal of Cerebral Blood Flow and Metabolism: Official Journal of the International Society of Cerebral Blood Flow and Metabolism, 38(9), 1418–1437. 10.1177/0271678x17702156 28393659 PMC6120130

[IMAG.a.964-b14] Clement, P., Petr, J., Dijsselhof, M. B. J., Padrela, B., Pasternak, M., Dolui, S., Jarutyte, L., Pinter, N., Hernandez-Garcia, L., Jahn, A., Kuijer, J. P. A., Barkhof, F., Mutsaerts, H. J. M. M., & Keil, V. C. (2022). A Beginner’s Guide to Arterial Spin Labeling (ASL) image processing. Frontiers in Radiology, 2, 929533. 10.3389/fradi.2022.929533 37492666 PMC10365107

[IMAG.a.964-b15] Cole, J. H., Ritchie, S. J., Bastin, M. E., Valdés Hernández, M. C., Muñoz Maniega, S., Royle, N., Corley, J., Pattie, A., Harris, S. E., Zhang, Q., Wray, N. R., Redmond, P., Marioni, R. E., Starr, J. M., Cox, S. R., Wardlaw, J. M., Sharp, D. J., & Deary, I. J. (2018). Brain age predicts mortality. Molecular Psychiatry, 23(5), 1385–1392. 10.1038/mp.2017.62 28439103 PMC5984097

[IMAG.a.964-b16] Da-Ano, R., Lucia, F., Masson, I., Abgral, R., Alfieri, J., Rousseau, C., Mervoyer, A., Reinhold, C., Pradier, O., Schick, U., Visvikis, D., & Hatt, M. (2021). A transfer learning approach to facilitate ComBat-based harmonization of multicentre radiomic features in new datasets. PLoS One, 16(7), e0253653. 10.1371/journal.pone.0253653 34197503 PMC8248970

[IMAG.a.964-b17] Damestani, N. L., Jacoby, J., Yadav, S. M., Lovely, A. E., Michael, A., Terpstra, M., Eshghi, M., Rashid, B., Cruchaga, C., Salat, D. H., & Juttukonda, M. R. (2023). Associations between age, sex, APOE genotype, and regional vascular physiology in typically aging adults. NeuroImage, 275, 120167. 10.1016/j.neuroimage.2023.120167 37187365 PMC10339339

[IMAG.a.964-b18] de Lange, A.-M. G., Anatürk, M., Rokicki, J., Han, L. K. M., Franke, K., Alnaes, D., Ebmeier, K. P., Draganski, B., Kaufmann, T., Westlye, L. T., Hahn, T., & Cole, J. H. (2022). Mind the gap: Performance metric evaluation in brain-age prediction. Human Brain Mapping, 43(10), 3113–3129. 10.1002/hbm.25837 35312210 PMC9188975

[IMAG.a.964-b19] de Lange, A.-M. G., & Cole, J. H. (2020). Commentary: Correction procedures in brain-age prediction [Review of *Commentary: Correction procedures in brain-age prediction*]. NeuroImage. Clinical, 26, 102229. 10.1016/j.nicl.2020.102229 32120292 PMC7049655

[IMAG.a.964-b20] Dijsselhof, M. B. J., Barboure, M., Stritt, M., Nordhøy, W., Wink, A. M., Beck, D., Westlye, L. T., Cole, J. H., Barkhof, F., Mutsaerts, H. J. M. M., & Petr, J. (2023). The value of arterial spin labelling perfusion MRI in brain age prediction. Human Brain Mapping, 44(7), 2754–2766. 10.1002/hbm.26242 36852443 PMC10089088

[IMAG.a.964-b21] Dijsselhof, M. B. J., Holtrop, J., James, S.-N., Sudre, C. H., Lu, K., Lorenzini, L., Collij, L. E., Scott, C. J., Manning, E. N., Thomas, D. L., Richards, M., Hughes, A. D., Cash, D. M., Barkhof, F., Schott, J. M., Petr, J., & Mutsaerts, H. J. (2025). Associations of life-course cardiovascular risk factors with late-life cerebral haemodynamics. Journal of Cerebral Blood Flow and Metabolism: Official Journal of the International Society of Cerebral Blood Flow and Metabolism, 45(4), 765–778. 10.1177/0271678x241301261 39552078 PMC11571377

[IMAG.a.964-b22] Dolui, S., Detre, J. A., Gaussoin, S. A., Herrick, J. S., Wang, D. J. J., Tamura, M. K., Cho, M. E., Haley, W. E., Launer, L. J., Punzi, H. A., Rastogi, A., Still, C. H., Weiner, D. E., Wright, J. T., Jr, Williamson, J. D., Wright, C. B., Bryan, R. N., Bress, A. P., Pajewski, N. M., & Nasrallah, I. M. (2022). Association of intensive vs standard blood pressure control with cerebral blood flow: Secondary analysis of the SPRINT MIND randomized clinical trial. JAMA Neurology, 79(4), 380–389. 10.1001/jamaneurol.2022.0074 35254390 PMC8902686

[IMAG.a.964-b23] Falcon, C., Montesinos, P., Václavu˚ , L., Kassinopoulos, M., Minguillon, C., Fauria, K., Cascales-Lahoz, D., Contador, J., Fernández-Lebrero, A., Navalpotro, I., Puig-Pijoan, A., Grau-Rivera, O., Kollmorgen, G., Quijano-Rubio, C., Molinuevo, J. L., Zetterberg, H., Blennow, K., Suárez-Calvet, M., Van Osch, M. J. P., … Gispert, J. D. (2024). Time-encoded ASL reveals lower cerebral blood flow in the early AD continuum. Alzheimer’s & Dementia: The Journal of the Alzheimer’s Association, 20(8), 5183–5197. 10.1002/alz.14059 PMC11350027 38958557

[IMAG.a.964-b24] Fortin, J.-P., Parker, D., Tunç, B., Watanabe, T., Elliott, M. A., Ruparel, K., Roalf, D. R., Satterthwaite, T. D., Gur, R. C., Gur, R. E., Schultz, R. T., Verma, R., & Shinohara, R. T. (2017). Harmonization of multi-site diffusion tensor imaging data. NeuroImage, 161, 149–170. 10.1016/j.neuroimage.2017.08.047 28826946 PMC5736019

[IMAG.a.964-b25] Franke, K., Bublak, P., Hoyer, D., Billiet, T., Gaser, C., Witte, O. W., & Schwab, M. (2020). In vivo biomarkers of structural and functional brain development and aging in humans. Neuroscience and Biobehavioral Reviews, 117, 142–164. 10.1016/j.neubiorev.2017.11.002 33308708

[IMAG.a.964-b200] Gaser, C. (2009). Partial volume segmentation with adaptive maximum a posteriori (MAP) approach. NeuroImage, 47(Supplement 1), S121. 10.1016/S1053-8119(09)71151-6

[IMAG.a.964-b26] Gaser, C., Kalc, P., & Cole, J. H. (2024). A perspective on brain-age estimation and its clinical promise. Nature Computational Science, 4(10), 744–751. 10.1038/s43588-024-00659-8 39048692

[IMAG.a.964-b27] Grade, M., Hernandez Tamames, J. A., Pizzini, F. B., Achten, E., Golay, X., & Smits, M. (2015). A neuroradiologist’s guide to arterial spin labeling MRI in clinical practice. Neuroradiology, 57(12), 1181–1202. 10.1007/s00234-015-1571-z 26351201 PMC4648972

[IMAG.a.964-b28] Graff, B. J., Harrison, S. L., Payne, S. J., & El-Bouri, W. K. (2023). Regional cerebral blood flow changes in healthy ageing and Alzheimer’s disease: A narrative review. Cerebrovascular Diseases (Basel, Switzerland), 52(1), 11–20. 10.1159/000524797 35640565

[IMAG.a.964-b29] Gyanwali, B., Tan, C. S., Escobosa, L. L. T., Vrooman, H. A., Chen, C., Mutsaerts, H. J. M. M., & Hilal, S. (2021). Determinants of arterial spin labeling parameters and its association with cerebral small vessel disease and diagnostic groups. Alzheimer’s & Dementia: The Journal of the Alzheimer’s Association, 17(Suppl. 4), e054701. 10.1002/alz.054701

[IMAG.a.964-b30] Hafdi, M., Mutsaerts, H. J., Petr, J., Richard, E., & van Dalen, J. W. (2022). Atherosclerotic risk is associated with cerebral perfusion—A cross-sectional study using arterial spin labeling MRI. NeuroImage. Clinical, 36, 103142. 10.1016/j.nicl.2022.103142 35970112 PMC9400119

[IMAG.a.964-b31] Horng, H., Singh, A., Yousefi, B., Cohen, E. A., Haghighi, B., Katz, S., Noël, P. B., Shinohara, R. T., & Kontos, D. (2022). Generalized ComBat harmonization methods for radiomic features with multi-modal distributions and multiple batch effects. Scientific Reports, 12(1), 4493. 10.1038/s41598-022-08412-9 35296726 PMC8927332

[IMAG.a.964-b32] Hu, F., Chen, A. A., Horng, H., Bashyam, V., Davatzikos, C., Alexander-Bloch, A., Li, M., Shou, H., Satterthwaite, T. D., Yu, M., & Shinohara, R. T. (2023). Image harmonization: A review of statistical and deep learning methods for removing batch effects and evaluation metrics for effective harmonization. NeuroImage, 274, 120125. 10.1016/j.neuroimage.2023.120125 37084926 PMC10257347

[IMAG.a.964-b33] Iadecola, C., & Gottesman, R. F. (2019). Neurovascular and cognitive dysfunction in hypertension. Circulation Research, 124(7), 1025–1044. 10.1161/circresaha.118.313260 30920929 PMC6527115

[IMAG.a.964-b34] Iturria-Medina, Y., Sotero, R. C., Toussaint, P. J., Mateos-Pérez, J. M., Evans, A. C., & Alzheimer’s Disease Neuroimaging Initiative. (2016). Early role of vascular dysregulation on late-onset Alzheimer’s disease based on multifactorial data-driven analysis. Nature Communications, 7, 11934. 10.7554/elife.62589 PMC4919512 27327500

[IMAG.a.964-b35] Iutaka, T., de Freitas, M. B., Omar, S. S., Scortegagna, F. A., Nael, K., Nunes, R. H., Pacheco, F. T., Maia Júnior, A. C. M., do Amaral, L. L. F., & da Rocha, A. J. (2023). Arterial spin labeling: Techniques, clinical applications, and interpretation. Radiographics: A Review Publication of the Radiological Society of North America, Inc, 43(1), e220088. 10.1148/rg.220088 36367822

[IMAG.a.964-b36] James, S.-N., Lane, C. A., Parker, T. D., Lu, K., Collins, J. D., Murray-Smith, H., Byford, M., Wong, A., Keshavan, A., Buchanan, S., Keuss, S. E., Kuh, D., Fox, N. C., Schott, J. M., & Richards, M. (2018). Using a birth cohort to study brain health and preclinical dementia: Recruitment and participation rates in Insight 46. BMC Research Notes, 11(1), 885. 10.1186/s13104-018-3995-0 30545411 PMC6293512

[IMAG.a.964-b37] Jirsaraie, R. J., Gorelik, A. J., Gatavins, M. M., Engemann, D. A., Bogdan, R., Barch, D. M., & Sotiras, A. (2023). A systematic review of multimodal brain age studies: Uncovering a divergence between model accuracy and utility. Patterns (New York, N.Y.), 4(4), 100712. 10.1016/j.patter.2023.100712 37123443 PMC10140612

[IMAG.a.964-b38] Jones, S., Tillin, T., Park, C., Williams, S., Rapala, A., Al Saikhan, L., Eastwood, S. V., Richards, M., Hughes, A. D., & Chaturvedi, N. (2020). Cohort profile update: Southall and Brent Revisited (SABRE) study: A UK population-based comparison of cardiovascular disease and diabetes in people of European, South Asian and African Caribbean heritage. International Journal of Epidemiology, 49(5), 1441e–1442e. 10.1093/ije/dyaa135 33049759 PMC7746410

[IMAG.a.964-b39] Lane, C. A., Parker, T. D., Cash, D. M., Macpherson, K., Donnachie, E., Murray-Smith, H., Barnes, A., Barker, S., Beasley, D. G., Bras, J., Brown, D., Burgos, N., Byford, M., Jorge Cardoso, M., Carvalho, A., Collins, J., De Vita, E., Dickson, J. C., Epie, N., … Schott, J. M. (2017). Study protocol: Insight 46—A neuroscience sub-study of the MRC National Survey of Health and Development. BMC Neurology, 17(1), 75. 10.1186/s12883-017-0846-x 28420323 PMC5395844

[IMAG.a.964-b40] Lindner, T., Bolar, D. S., Achten, E., Barkhof, F., Bastos-Leite, A. J., Detre, J. A., Golay, X., Günther, M., Wang, D. J. J., Haller, S., Ingala, S., Jäger, H. R., Jahng, G.-H., Juttukonda, M. R., Keil, V. C., Kimura, H., Ho, M.-L., Lequin, M., Lou, X.,…on behalf of the ISMRM Perfusion Study Group. (2023). Current state and guidance on arterial spin labeling perfusion MRI in clinical neuroimaging. Magnetic Resonance in Medicine: Official Journal of the Society of Magnetic Resonance in Medicine / Society of Magnetic Resonance in Medicine, 89(5), 2024–2047. 10.1002/mrm.29572 PMC10914350 36695294

[IMAG.a.964-b41] Lombardi, A., Amoroso, N., Diacono, D., Monaco, A., Tangaro, S., & Bellotti, R. (2020). Extensive evaluation of morphological statistical harmonization for brain age prediction. Brain Sciences, 10(6), 364. 10.3390/brainsci10060364 32545374 PMC7349402

[IMAG.a.964-b42] Maikusa, N., Zhu, Y., Uematsu, A., Yamashita, A., Saotome, K., Okada, N., Kasai, K., Okanoya, K., Yamashita, O., Tanaka, S. C., & Koike, S. (2021). Comparison of traveling-subject and ComBat harmonization methods for assessing structural brain characteristics. Human Brain Mapping, 42(16), 5278–5287. 10.1002/hbm.25615 34402132 PMC8519865

[IMAG.a.964-b43] Marzi, C., Giannelli, M., Barucci, A., Tessa, C., Mascalchi, M., & Diciotti, S. (2024). Efficacy of MRI data harmonization in the age of machine learning: A multicenter study across 36 datasets. Scientific Data, 11(1), 115. 10.1038/s41597-023-02421-7 38263181 PMC10805868

[IMAG.a.964-b44] Morgan, C. A., Melzer, T. R., Roberts, R. P., Wiebels, K., Mutsaerts, H. J. M. M., Spriggs, M. J., Dalrymple-Alford, J. C., Anderson, T. J., Cutfield, N. J., Deib, G., Pfeuffer, J., Addis, D. R., Kirk, I. J., & Tippett, L. J. (2021). Spatial variation of perfusion MRI reflects cognitive decline in mild cognitive impairment and early dementia. Scientific Reports, 11(1), 23325. 10.1038/s41598-021-02313-z 34857793 PMC8639710

[IMAG.a.964-b201] Mutsaerts, H. J. M. M., Petr, J., Groot, P., Vandemaele, P., Ingala, S., Robertson, A. D., Václavů, L., Groote, I., Kuijf, H., Zelaya, F., O’Daly, O., Hila, S., Wink, A. M., Kant, I., Caan, M. W. A., Morgan, C., de Bresser, J., Lysvik, E., Schrantee, A., Bjørnebekk, A. … Barkhof, F. (2020). ExploreASL: An image processing pipeline for multi-center ASL perfusion MRI studies. *NeuroImage*, 219, 117031. 10.1016/j.neuroimage.2020.117031 32526385

[IMAG.a.964-b45] Mutsaerts, H. J. M. M., Petr, J., Václavu˚ , L., van Dalen, J. W., Robertson, A. D., Caan, M. W., Masellis, M., Nederveen, A. J., Richard, E., & MacIntosh, B. J. (2017). The spatial coefficient of variation in arterial spin labeling cerebral blood flow images. Journal of Cerebral Blood Flow and Metabolism: Official Journal of the International Society of Cerebral Blood Flow and Metabolism, 37(9), 3184–3192. 10.1177/0271678x16683690 28058975 PMC5584689

[IMAG.a.964-b46] Mutsaerts, H. J. M. M., Steketee, R. M. E., Heijtel, D. F. R., Kuijer, J. P. A., van Osch, M. J. P., Majoie, C. B. L. M., Smits, M., & Nederveen, A. J. (2014). Inter-vendor reproducibility of pseudo-continuous arterial spin labeling at 3 Tesla. PLoS One, 9(8), e104108. 10.1371/journal.pone.0104108 25090654 PMC4121318

[IMAG.a.964-b47] Mutsaerts, H. J. M. M., van Osch, M. J. P., Zelaya, F. O., Wang, D. J. J., Nordhøy, W., Wang, Y., Wastling, S., Fernandez-Seara, M. A., Petersen, E. T., Pizzini, F. B., Fallatah, S., Hendrikse, J., Geier, O., Günther, M., Golay, X., Nederveen, A. J., Bjørnerud, A., & Groote, I. R. (2015). Multi-vendor reliability of arterial spin labeling perfusion MRI using a near-identical sequence: Implications for multi-center studies. NeuroImage, 113, 143–152. 10.1016/j.neuroimage.2015.03.043 25818685

[IMAG.a.964-b48] Pantoni, L. (2010). Cerebral small vessel disease: From pathogenesis and clinical characteristics to therapeutic challenges. Lancet Neurology, 9(7), 689–701. 10.1016/s1474-4422(10)70104-6 20610345

[IMAG.a.964-b49] Paschoal, A. M., Woods, J. G., Pinto, J., Bron, E. E., Petr, J., Kennedy McConnell, F. A., Bell, L., Dounavi, M.-E., van Praag, C. G., Mutsaerts, H. J. M. M., Taylor, A. O., Zhao, M. Y., Brumer, I., Chan, W. S. M., Toner, J., Hu, J., Zhang, L. X., Domingos, C., Monteiro, S. P., … Anazodo, U. (2024). Reproducibility of arterial spin labeling cerebral blood flow image processing: A report of the ISMRM open science initiative for perfusion imaging (OSIPI) and the ASL MRI challenge. Magnetic Resonance in Medicine, 92(2), 836–852. 10.1002/mrm.30081 38502108 PMC11497242

[IMAG.a.964-b50] Pomponio, R., Erus, G., Habes, M., Doshi, J., Srinivasan, D., Mamourian, E., Bashyam, V., Nasrallah, I. M., Satterthwaite, T. D., Fan, Y., Launer, L. J., Masters, C. L., Maruff, P., Zhuo, C., Völzke, H., Johnson, S. C., Fripp, J., Koutsouleris, N., Wolf, D. H., … Davatzikos, C. (2020). Harmonization of large MRI datasets for the analysis of brain imaging patterns throughout the lifespan. NeuroImage, 208, 116450. 10.1016/j.neuroimage.2019.116450 31821869 PMC6980790

[IMAG.a.964-b202] R Core Team (2023). The R project for statistical computing. https://www.R-project.org/

[IMAG.a.964-b51] Rokicki, J., Wolfers, T., Nordhøy, W., Tesli, N., Quintana, D. S., Alnaes, D., Richard, G., de Lange, A.-M. G., Lund, M. J., Norbom, L., Agartz, I., Melle, I., Naerland, T., Selbaek, G., Persson, K., Nordvik, J. E., Schwarz, E., Andreassen, O. A., Kaufmann, T., & Westlye, L. T. (2021). Multimodal imaging improves brain age prediction and reveals distinct abnormalities in patients with psychiatric and neurological disorders. Human Brain Mapping, 42(6), 1714–1726. 10.1002/hbm.25323 33340180 PMC7978139

[IMAG.a.964-b52] Rozemberczki, B., Watson, L., Bayer, P., Yang, H.-T., Kiss, O., Nilsson, S., & Sarkar, R. (2022). The Shapley Value in Machine Learning. arXiv [cs.LG]. http://arxiv.org/abs/2202.05594

[IMAG.a.964-b53] Schmidt, P., Gaser, C., Arsic, M., Buck, D., Förschler, A., Berthele, A., Hoshi, M., Ilg, R., Schmid, V. J., Zimmer, C., Hemmer, B., & Mühlau, M. (2012). An automated tool for detection of FLAIR-hyperintense white-matter lesions in Multiple Sclerosis. NeuroImage, 59(4), 3774–3783. 10.1016/j.neuroimage.2011.11.032 22119648

[IMAG.a.964-b54] Snijder, M. B., Galenkamp, H., Prins, M., Derks, E. M., Peters, R. J. G., Zwinderman, A. H., & Stronks, K. (2017). Cohort profile: The Healthy Life in an Urban Setting (HELIUS) study in Amsterdam, The Netherlands. BMJ Open, 7(12), e017873. 10.1136/bmjopen-2017-017873 PMC5736025 29247091

[IMAG.a.964-b55] Tatu, L., Moulin, T., Vuillier, F., & Bogousslavsky, J. (2012). Arterial territories of the human brain. Frontiers of Neurology and Neuroscience, 30, 99–110. 10.1159/000333602 22377874

[IMAG.a.964-b56] Tryambake, D., He, J., Firbank, M. J., O’Brien, J. T., Blamire, A. M., & Ford, G. A. (2013). Intensive blood pressure lowering increases cerebral blood flow in older subjects with hypertension. Hypertension, 61(6), 1309–1315. 10.1161/hypertensionaha.112.200972 23529166

[IMAG.a.964-b57] van Dinther, M., Hooghiemstra, A. M., Bron, E. E., Versteeg, A., Leeuwis, A. E., Kalay, T., Moonen, J. E., Kuipers, S., Backes, W. H., Jansen, J. F. A., van Osch, M. J. P., Biessels, G.-J., Staals, J., van Oostenbrugge, R. J., & Heart-Brain Connection consortium. (2024). Lower cerebral blood flow predicts cognitive decline in patients with vascular cognitive impairment. Alzheimer’s & Dementia: The Journal of the Alzheimer’s Association, 20(1), 136–144. 10.1002/alz.13408 PMC10917014 37491840

[IMAG.a.964-b58] van Nederpelt, D. R., Amiri, H., Brouwer, I., Noteboom, S., Mokkink, L. B., Barkhof, F., Vrenken, H., & Kuijer, J. P. A. (2023). Reliability of brain atrophy measurements in multiple sclerosis using MRI: An assessment of six freely available software packages for cross-sectional analyses. Neuroradiology, 65(10), 1459–1472. 10.1007/s00234-023-03189-8 37526657 PMC10497452

[IMAG.a.964-b59] van Osch, M. J., Teeuwisse, W. M., Chen, Z., Suzuki, Y., Helle, M., & Schmid, S. (2018). Advances in arterial spin labelling MRI methods for measuring perfusion and collateral flow. Journal of Cerebral Blood Flow and Metabolism: Official Journal of the International Society of Cerebral Blood Flow and Metabolism, 38(9), 1461–1480. 10.1177/0271678x17713434 28598243 PMC6120125

[IMAG.a.964-b60] Wagen, A. Z., Coath, W., Keshavan, A., James, S.-N., Parker, T. D., Lane, C. A., Buchanan, S. M., Keuss, S. E., Storey, M., Lu, K., Macdougall, A., Murray-Smith, H., Freiberger, T., Cash, D. M., Malone, I. B., Barnes, J., Sudre, C. H., Wong, A., Pavisic, I. M., … Schott, J. M. (2022). Life course, genetic, and neuropathological associations with brain age in the 1946 British Birth Cohort: A population-based study. The Lancet. Healthy Longevity, 3(9), e607–e616. 10.1016/s2666-7568(22)00167-2 36102775 PMC10499760

[IMAG.a.964-b61] Williamson, W., Lewandowski, A. J., Forkert, N. D., Griffanti, L., Okell, T. W., Betts, J., Boardman, H., Siepmann, T., McKean, D., Huckstep, O., Francis, J. M., Neubauer, S., Phellan, R., Jenkinson, M., Doherty, A., Dawes, H., Frangou, E., Malamateniou, C., Foster, C., & Leeson, P. (2018). Association of cardiovascular risk factors with MRI indices of cerebrovascular structure and function and white matter hyperintensities in young adults. JAMA: The Journal of the American Medical Association, 320(7), 665–673. 10.1001/jama.2018.11498 30140877 PMC6142949

[IMAG.a.964-b62] Wong, L. C. K., Wong, M. Y. Z., Tan, C. S., Vrooman, H., Venketasubramanian, N., Cheng, C.-Y., Chen, C., & Hilal, S. (2020). Interethnic differences in neuroimaging markers and cognition in Asians, a population-based study. Scientific Reports, 10(1), 2655. 10.1038/s41598-020-59618-8 32060376 PMC7021682

[IMAG.a.964-b63] Zhang, R., Oliver, L. D., Voineskos, A. N., & Park, J. Y. (2023). RELIEF: A structured multivariate approach for removal of latent inter-scanner effects. Imaging Neuroscience (Cambridge, Mass.), 1, 1–16. 10.1162/imag_a_00011 PMC10503485 37719839

