# Supplementary material for: Multi-cohort, multi-sequence harmonisation for cerebrovascular brain age
Source: Imaging Neurosci (Camb). 2025 Oct 27;3:IMAG.a.964. doi: 10.1162/IMAG.a.964 (PMC12559958; doi:10.1162/IMAG.a.964)
Supplement: Supplementary Material [file IMAG.a.964_supp.pdf]

Supplementary material

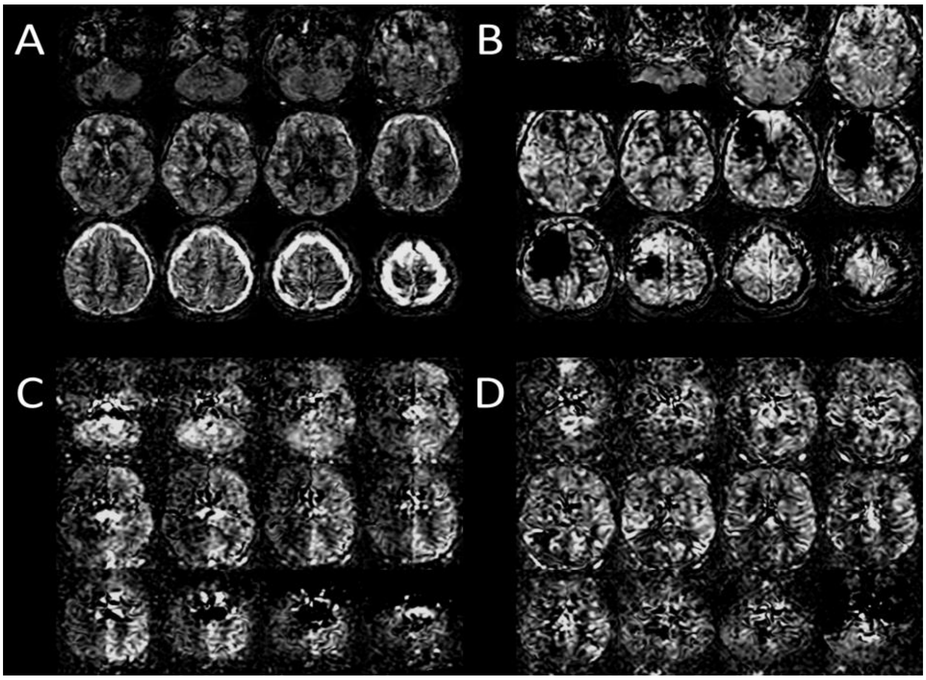

Supplementary Figure 1: Examples of excluded CBF maps, showing motion artefacts (A), possible coil issues (B), asymmetric labelling efficiency (C), hyperintense vascular signal (C,D), and arterial transit artefact-related hypointense signal (D). *CBF: cerebral blood flow.*

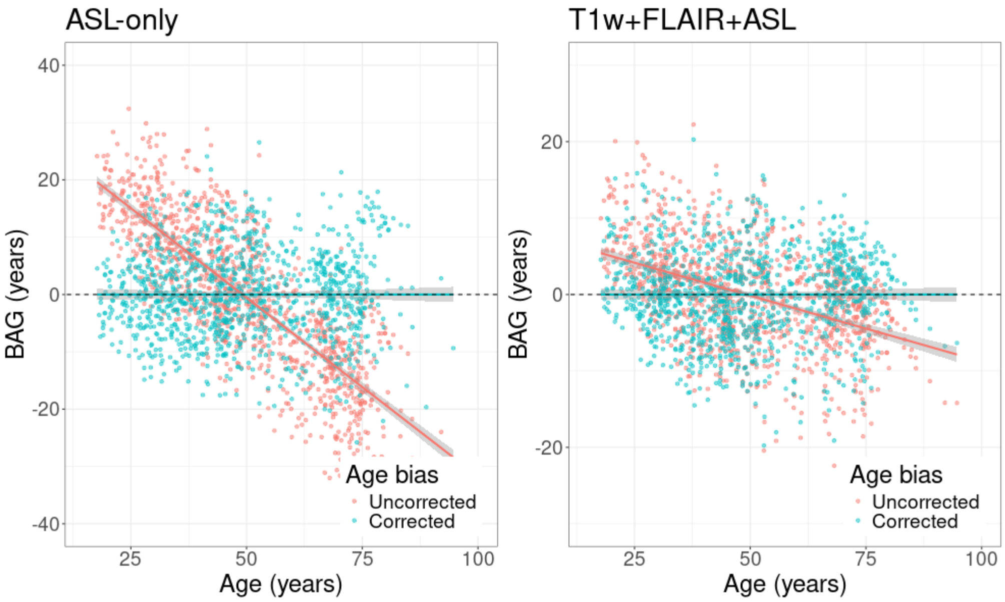

Supplementary Figure 2: The association of the validation dataset uncorrected (red) and age bias-corrected (blue) BAGs with the chronological age of the ASL-only and T1w+FLAIR+ASL models before harmonisation. The dashed black line represents a perfect estimation. *ASL: arterial spin labelling; BAG: Brain age gap; FLAIR: Fluid attenuated inversion recovery; T1w: T1-weighted.*

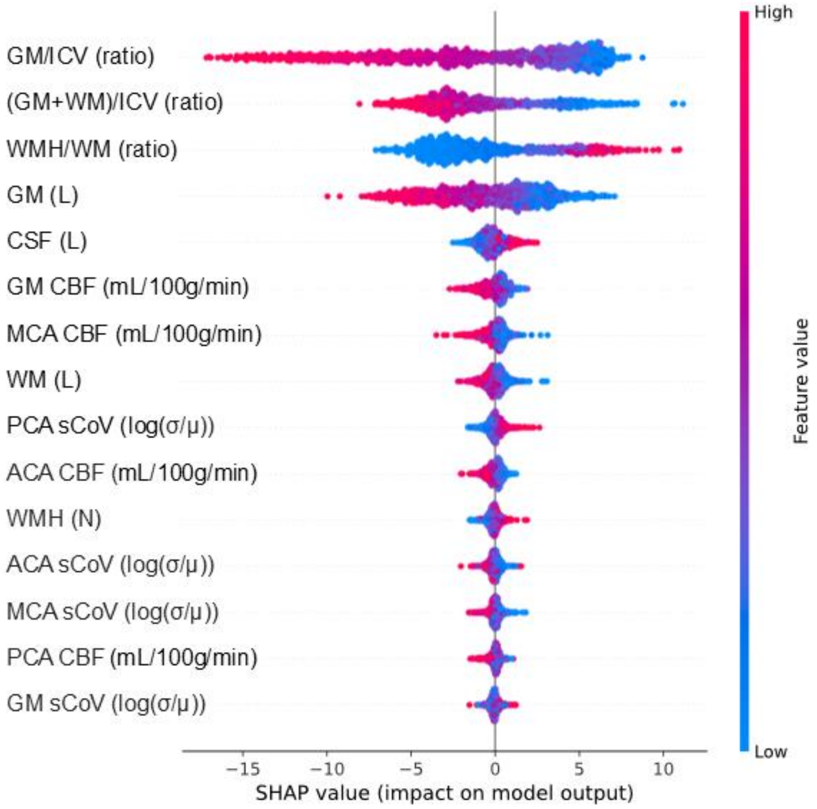

Supplementary Figure 3: Feature importance of the T1w+FLAIR+ASL brain age estimation model derived using Shapley values. *ACA: anterior cerebral artery; CBF: cerebral blood flow; CSF: cerebrospinal fluid; GM: grey matter; ICV: intracranial volume; MCA: middle cerebral artery; PCA: posterior cerebral artery; sCoV: spatial coefficient of variation; WM: white matter; WMH: white matter hyperintensities.*

Supplementary Table 1: ANCOVA with Tukey post hoc test results, corrected for age and sex, of differences in unharmonised imaging feature between cohort-pairs, shown in mean [confidence interval]. WMH/WM, WMH count, and all sCoV features have been log-transformed. *ACA: anterior cerebral artery; CBF: cerebral blood flow; CSF: cerebrospinal fluid; GM: grey matter; ICV: intracranial volume; MCA: middle cerebral artery; PCA: posterior cerebral artery; sCoV: spatial coefficient of variation; WM: white matter; WMH: white matter hyperintensities.*

**Feature**	**EDIS - HELIUS**	**EDIS - Insight46**	**EDIS - SABRE**	**EDIS - Training**	**HELIUS - Insight46**	**HELIUS - SABRE**	**HELIUS - Training**	**Insight46 - SABRE**	**Insight46 - Training**	**SABRE - Training**
**GM (L)**										
Difference	-0.04	-0.06	0	-0.11	-0.02	0.04	-0.07	0.06	-0.05	-0.11
	[-0.05, -0.03]	[-0.07, -0.06]	[-0.01, 0.00]	[-0.12, -0.10]	[-0.03, -0.02]	[0.03, 0.04]	[-0.08, -0.06]	[0.05, 0.07]	[-0.05, -0.04]	[-0.11, -0.10]
P-value	<0.001	<0.001	0.939	<0.001	<0.001	<0.001	<0.001	<0.001	<0.001	<0.001
**WM (L)**										
Difference	-0.08	-0.06	-0.02	-0.10	0.02	0.06	-0.02	0.04	-0.04	-0.08
	[-0.09, -0.07]	[-0.07, -0.05]	[-0.03, -0.01]	[-0.11, -0.1]	[0.01, 0.03]	[0.05, 0.07]	[-0.03, -0.02]	[0.03, 0.04]	[-0.05, -0.04]	[-0.09, -0.07]
P-value	<0.001	<0.001	<0.001	<0.001	<0.001	<0.001	<0.001	<0.001	<0.001	<0.001
**CSF (L)**										
Difference	-0.07	-0.01	0.01	-0.10	0.06	0.08	-0.04	0.02	-0.10	-0.12
	[-0.08, -0.06]	[-0.02, 0.00]	[0.00, 0.02]	[-0.11, -0.09]	[0.05, 0.07]	[0.07, 0.09]	[-0.04, -0.03]	[0.01, 0.03]	[-0.11, -0.09]	[-0.13, -0.11]
P-value	<0.001	0.785	0.021	<0.001	<0.001	<0.001	<0.001	<0.001	<0.001	<0.001
**GM/ICV (ratio)**										
Difference	0.02	-0.01	0.00	0.01	-0.03	-0.02	-0.01	0.01	0.02	0.01
	[0.02, 0.03]	[-0.01, -0.01]	[0.00, 0.00]	[0.01, 0.02]	[-0.04, -0.03]	[-0.03, -0.02]	[-0.01, -0.01]	[0.01, 0.01]	[0.02, 0.03]	[0.01, 0.02]
P-value	<0.001	<0.001	0.956	<0.001	<0.001	<0.001	<0.001	<0.001	<0.001	<0.001
**(GM+WM)/ICV ratio**										
Difference	0.01	-0.02	-0.01	0.02	-0.03	-0.02	0.00	0.01	0.04	0.03
	[0.01, 0.02]	[-0.03, -0.01]	[-0.02, -0.01]	[0.01, 0.02]	[-0.04, -0.03]	[-0.03, -0.02]	[0.00, 0.01]	[0.00, 0.01]	[0.03, 0.04]	[0.02, 0.03]
P-value	<0.001	<0.001	<0.001	<0.001	<0.001	<0.001	0.230	0.033	<0.001	<0.001

Feature	EDIS - HELIUS	EDIS - Insight46	EDIS - SABRE	EDIS - Training	HELIUS - Insight 46	HELIUS - SABRE	HELIUS - Training	Insight 46 - SABRE	Insight 46 - Training	SABRE - Training
**WMH/WM (ratio)**										
Difference	0.00	0.01	0.00	-0.01	0.01	0.00	-0.01	-0.01	-0.02	-0.02
	[0.00, 0.00]	[0.01, 0.01]	[0.00, 0.01]	[-0.01, -0.01]	[0.01, 0.02]	[0.00, 0.01]	[-0.01, -0.01]	[-0.01, -0.01]	[-0.03, -0.02]	[-0.02, -0.01]
P-value	0.688	<0.001	0.020	<0.001	<0.001	<0.001	<0.001	<0.001	<0.001	<0.001
**WMH (N)**										
Difference	-0.28	0.05	-0.03	-0.40	0.33	0.25	-0.12	-0.08	-0.45	-0.37
	[-0.34, -0.21]	[-0.02, 0.12]	[-0.09, 0.03]	[-0.46, -0.33]	[0.26, 0.40]	[0.19, 0.30]	[-0.17, -0.08]	[-0.14, -0.02]	[-0.52, -0.38]	[-0.43, -0.31]
P-value	<0.001	0.554	0.866	<0.001	<0.001	<0.001	<0.001	0.068	<0.001	<0.001
**GM CBF (mL/100g/min)**										
Difference	15.84	10.69	23.94	13.89	-5.15	8.10	-1.96	13.25	3.20	-10.05
	[14.16, 17.53]	[8.88, 12.5]	[22.44, 25.45]	[12.19, 15.58]	[-6.95, -3.36]	[6.60, 9.59]	[-3.20, -0.71]	[11.64, 14.86]	[1.38, 5.01]	[-11.58, -8.53]
P-value	<0.001	<0.001	<0.001	<0.001	<0.001	<0.001	0.018	<0.001	0.005	<0.001
**ACA CBF (mL/100g/min)**										
Difference	10.52	5.27	23.57	7.96	-5.25	13.05	-2.56	18.29	2.69	-15.61
	[8.45, 12.59]	[3.05, 7.50]	[21.72, 25.42]	[5.87, 10.04]	[-7.46, -3.04]	[11.21, 14.89]	[-4.09, -1.03]	[16.32, 20.27]	[0.45, 4.92]	[-17.48, -13.73]
P-value	<0.001	<0.001	<0.001	<0.001	<0.001	<0.001	<0.001	<0.001	0.128	<0.001
**MCA CBF (mL/100g/min)**										
Difference	18.35	9.80	30.71	15.71	-8.55	12.36	-2.64	20.90	5.90	-15.00
	[16.46, 20.24]	[7.77, 11.83]	[29.02, 32.39]	[13.80, 17.61]	[-10.56, -6.53]	[10.68, 14.04]	[-4.04, -1.25]	[19.1, 22.71]	[3.86, 7.94]	[-16.71, -13.29]
P-value	<0.001	<0.001	<0.001	<0.001	<0.001	<0.001	0.002	<0.001	<0.001	<0.001
**PCA CBF (mL/100g/min)**										
Difference	12.69	5.50	18.54	11.08	-7.19	5.84	-1.62	13.03	5.58	-7.46
	[10.97, 14.42]	[3.65, 7.36]	[16.99, 20.08]	[9.34, 12.82]	[-9.03, -5.35]	[4.30, 7.38]	[-2.89, -0.34]	[11.38, 14.68]	[3.71, 7.44]	[-9.02, -5.89]
P-value	<0.001	<0.001	<0.001	<0.001	<0.001	<0.001	0.095	<0.001	<0.001	<0.001

Feature	EDIS - HELIUS	EDIS - Insight46	EDIS - SABRE	EDIS - Training	HELIUS - Insight 46	HELIUS - SABRE	HELIUS - Training	Insight 46 - SABRE	Insight 46 - Training	SABRE - Training
**GM sCoV (log(σ/μ))**										
Difference	0.40	0.37	0.26	0.92	-0.03	-0.15	0.52	-0.11	0.55	0.67
	[0.37, 0.43]	[0.34, 0.39]	[0.23, 0.28]	[0.89, 0.95]	[-0.06, -0.01]	[-0.17, -0.12]	[0.50, 0.54]	[-0.14, -0.09]	[0.53, 0.58]	[0.64, 0.69]
P-value	<0.001	<0.001	<0.001	<0.001	0.126	<0.001	<0.001	<0.001	<0.001	<0.001
**ACA sCoV (log(σ/μ))**										
Difference	0.40	0.28	0.21	0.99	-0.12	-0.19	0.58	-0.07	0.70	0.78
	[0.38, 0.43]	[0.25, 0.31]	[0.19, 0.24]	[0.96, 1.02]	[-0.15, -0.09]	[-0.22, -0.17]	[0.56, 0.6]	[-0.10, -0.05]	[0.67, 0.73]	[0.75, 0.8]
P-value	<0.001	<0.001	<0.001	<0.001	<0.001	<0.001	<0.001	<0.001	<0.001	<0.001
**MCA sCoV (log(σ/μ))**										
Difference	0.41	0.49	0.18	0.97	0.07	-0.23	0.56	-0.3	0.48	0.79
	[0.38, 0.44]	[0.46, 0.51]	[0.16, 0.21]	[0.94, 0.99]	[0.05, 0.10]	[-0.25, -0.21]	[0.54, 0.58]	[-0.33, -0.28]	[0.45, 0.51]	[0.76, 0.81]
P-value	<0.001	<0.001	<0.001	<0.001	<0.001	<0.001	<0.001	<0.001	<0.001	<0.001
**PCA sCoV (log(σ/μ))**										
Difference	0.51	0.49	0.42	1.09	-0.02	-0.09	0.59	-0.07	0.60	0.68
	[0.47, 0.54]	[0.46, 0.52]	[0.39, 0.44]	[1.06, 1.12]	[-0.05, 0.02]	[-0.12, -0.06]	[0.56, 0.61]	[-0.1, -0.04]	[0.57, 0.64]	[0.65, 0.71]
P-value	<0.001	<0.001	<0.001	<0.001	<0.001	<0.001	<0.001	<0.001	<0.001	<0.001

Supplementary Table 2: GM, ACA, MCA, and PCA CBF and log-transformed sCoV of the combined or separate testing cohort-pairs per harmonisation method. *ACA: anterior cerebral artery; CBF: cerebral blood flow; GM: grey matter; MCA: middle cerebral artery; PCA: posterior cerebral artery; sCoV: spatial coefficient of variation.*

Harmonisation method	GM CBF (mL/100g/min)	ACA CBF (mL/100g/min)	MCA CBF (mL/100g/min)	PCA CBF (mL/100g/min)	GM sCoV (log(σ/μ))	ACA sCoV (log(σ/μ))	MCA sCoV (log(σ/μ))	PCA sCoV (log(σ/μ))
Unharmonised	61.48 ± 14.46	75.88 ± 17.25	68.29 ± 16.87	54.69 ± 13.73	-1.04 ± 0.41	-1.17 ± 0.45	-1.08 ± 0.42	-1.08 ± 0.46

NeuroComBat	61.48 ± 12.66	75.88 ± 15.01	68.29 ± 13.97	54.69 ± 12.64	-1.04 ± 0.19	-1.17 ± 0.20	-1.08 ± 0.19	-1.08 ± 0.22
CovBat	61.48 ± 12.66	75.88 ± 15.01	68.29 ± 13.97	54.69 ± 12.65	-1.04 ± 0.19	-1.17 ± 0.20	-1.08 ± 0.19	-1.08 ± 0.22
NeuroHarmonize	61.45 ± 12.48	75.85 ± 14.93	68.27 ± 13.90	54.67 ± 12.43	-1.04 ± 0.19	-1.17 ± 0.20	-1.08 ± 0.19	-1.07 ± 0.22
OPN ComBat	61.40 ± 12.45	75.77 ± 14.87	68.20 ± 13.86	54.63 ± 12.41	-1.04 ± 0.19	-1.17 ± 0.20	-1.08 ± 0.19	-1.08 ± 0.22
AutoComBat	61.92 ± 12.83	76.19 ± 15.14	68.78 ± 14.24	55.1 ± 12.70	-1.04 ± 0.19	-1.17 ± 0.20	-1.08 ± 0.19	-1.09 ± 0.22
RELIEF	61.47 ± 10.98	75.86 ± 13.32	68.27 ± 12.16	54.68 ± 10.91	-1.04 ± 0.12	-1.17 ± 0.13	-1.08 ± 0.10	-1.08 ± 0.15

**Cohort**	**Harmonisation method**	**GM CBF (mL/100g/min)**	**ACA CBF (mL/100g/min)**	**MCA CBF (mL/100g/min)**	**PCA CBF (mL/100g/min)**	**GM sCoV (log(σ/μ))**	**ACA sCoV (log(σ/μ))**	**MCA sCoV (log(σ/μ))**	**PCA sCoV (log(σ/μ))**
Training	Unharmonised	66.2 ± 11.29	82.41 ± 13.34	73.74 ± 12.07	58.15 ± 10.64	-1.48 ± 0.11	-1.67 ± 0.12	-1.53 ± 0.08	-1.57 ± 0.13
	NeuroComBat	65.65 ± 12.98	79.76 ± 15.31	72.45 ± 14.26	58.17 ± 12.8	-1.07 ± 0.18	-1.2 ± 0.2	-1.09 ± 0.18	-1.12 ± 0.21
	CovBat	65.65 ± 12.98	79.76 ± 15.3	72.45 ± 14.26	58.17 ± 12.8	-1.07 ± 0.18	-1.2 ± 0.2	-1.09 ± 0.18	-1.12 ± 0.21
	NeuroHarmonize	65.33 ± 12.9	79.81 ± 15.26	72.19 ± 14.25	57.49 ± 12.71	-1.05 ± 0.19	-1.18 ± 0.2	-1.07 ± 0.18	-1.09 ± 0.21
	OPN ComBat	64.93 ± 12.83	79.29 ± 15.11	72.05 ± 14.07	57.07 ± 12.61	-1.06 ± 0.17	-1.19 ± 0.18	-1.09 ± 0.17	-1.12 ± 0.2
	AutoComBat	66.18 ± 13.45	80.23 ± 15.63	73.26 ± 14.8	58.33 ± 13.19	-1.08 ± 0.19	-1.19 ± 0.2	-1.09 ± 0.19	-1.14 ± 0.22
	RELIEF	61.48 ± 10.45	75.88 ± 12.42	68.29 ± 11.4	54.69 ± 9.86	-1.04 ± 0.11	-1.17 ± 0.11	-1.08 ± 0.08	-1.08 ± 0.13
HELIUS	Unharmonised	61.27 ± 9.88	77.07 ± 12.73	68.03 ± 11.3	54.27 ± 9.84	-0.94 ± 0.14	-1.07 ± 0.13	-0.97 ± 0.12	-0.95 ± 0.19
	NeuroComBat	62.71 ± 11.6	77.02 ± 14.17	69.42 ± 12.95	55.94 ± 12.06	-1.05 ± 0.19	-1.18 ± 0.2	-1.08 ± 0.19	-1.09 ± 0.23
	CovBat	62.71 ± 11.58	77.02 ± 14.13	69.42 ± 12.92	55.94 ± 12.05	-1.05 ± 0.19	-1.18 ± 0.2	-1.08 ± 0.19	-1.09 ± 0.23
	NeuroHarmonize	61.56 ± 11.53	75.87 ± 14.16	69.31 ± 12.95	55.22 ± 12.01	-1.06 ± 0.19	-1.19 ± 0.19	-1.09 ± 0.18	-1.09 ± 0.22
	OPN ComBat	61.61 ± 11.53	75.95 ± 14.05	68.46 ± 12.83	54.6 ± 12.02	-1.04 ± 0.19	-1.17 ± 0.19	-1.07 ± 0.18	-1.08 ± 0.23

	AutoComBat	62.59 ± 11.7	76.93 ± 14.22	69.4 ± 13.03	55.48 ± 12.2	-1.04 ± 0.2	-1.17 ± 0.2	-1.07 ± 0.19	-1.09 ± 0.23
	RELIEF	61.48 ± 10.06	75.88 ± 12.85	68.29 ± 11.28	54.69 ± 10.37	-1.04 ± 0.11	-1.17 ± 0.12	-1.08 ± 0.1	-1.08 ± 0.15
SABRE	Unharmonised	47.76 ± 11.47	58.98 ± 13.1	50.38 ± 12.32	43.63 ± 12.96	-0.74 ± 0.24	-0.83 ± 0.25	-0.71 ± 0.25	-0.8 ± 0.29
	NeuroComBat	57.26 ± 11.6	71.95 ± 14.22	64.1 ± 13.09	51.12 ± 11.89	-1 ± 0.19	-1.14 ± 0.2	-1.06 ± 0.19	-1.03 ± 0.22
	CovBat	57.26 ± 11.62	71.95 ± 14.25	64.1 ± 13.12	51.12 ± 11.9	-1 ± 0.19	-1.14 ± 0.2	-1.06 ± 0.19	-1.03 ± 0.22
	NeuroHarmonize	57.94 ± 11.52	72.3 ± 14.1	64.39 ± 12.98	52.21 ± 11.78	-1.02 ± 0.19	-1.15 ± 0.2	-1.07 ± 0.19	-1.06 ± 0.22
	OPN ComBat	57.92 ± 11.32	72.38 ± 13.88	64.44 ± 12.84	52.14 ± 11.67	-1.01 ± 0.19	-1.15 ± 0.2	-1.06 ± 0.19	-1.04 ± 0.22
	AutoComBat	57.2 ± 11.56	71.52 ± 14.15	63.8 ± 13.04	51.64 ± 11.87	-1.02 ± 0.19	-1.16 ± 0.2	-1.07 ± 0.19	-1.05 ± 0.22
	RELIEF	61.48 ± 8.93	75.88 ± 10.97	68.29 ± 9.9	54.69 ± 8.89	-1.04 ± 0.09	-1.17 ± 0.1	-1.08 ± 0.08	-1.08 ± 0.12
EDIS	Unharmonised	72.33 ± 15.25	83.15 ± 18.56	81.78 ± 17.39	62.59 ± 14.88	-0.49 ± 0.27	-0.63 ± 0.29	-0.53 ± 0.28	-0.4 ± 0.29
	NeuroComBat	57.9 ± 11.33	72.55 ± 13.97	64.79 ± 12.79	51.54 ± 11.64	-1 ± 0.18	-1.14 ± 0.2	-1.06 ± 0.19	-1.04 ± 0.22
	CovBat	57.9 ± 11.35	72.55 ± 14	64.79 ± 12.8	51.54 ± 11.68	-1 ± 0.18	-1.14 ± 0.2	-1.06 ± 0.19	-1.04 ± 0.22
	NeuroHarmonize	57.94 ± 11.3	72.26 ± 13.91	65.07 ± 12.68	52.38 ± 11.52	-1.03 ± 0.18	-1.16 ± 0.2	-1.08 ± 0.19	-1.07 ± 0.21
	OPN ComBat	59.56 ± 11.24	74.05 ± 13.82	66.11 ± 12.64	53.69 ± 11.51	-1.03 ± 0.19	-1.17 ± 0.2	-1.08 ± 0.19	-1.06 ± 0.21
	AutoComBat	59.18 ± 11.25	73.64 ± 13.83	65.86 ± 12.71	52.88 ± 11.43	-1.02 ± 0.19	-1.16 ± 0.2	-1.07 ± 0.19	-1.05 ± 0.21
	RELIEF	61.48 ± 14.54	75.88 ± 17.32	68.29 ± 16.19	54.69 ± 14.64	-1.04 ± 0.17	-1.17 ± 0.18	-1.08 ± 0.15	-1.08 ± 0.21
Insight46	Unharmonised	61.16 ± 14.79	77.42 ± 18.7	71.45 ± 17.84	56.76 ± 15.36	-0.86 ± 0.22	-0.91 ± 0.25	-1.01 ± 0.23	-0.88 ± 0.24
	NeuroComBat	57.16 ± 11.44	71.88 ± 14.14	64.2 ± 13.04	50.78 ± 11.67	-1 ± 0.18	-1.15 ± 0.19	-1.06 ± 0.18	-1.03 ± 0.21
	CovBat	57.16 ± 11.44	71.89 ± 14.12	64.2 ± 13.01	50.79 ± 11.66	-1 ± 0.18	-1.15 ± 0.19	-1.06 ± 0.18	-1.03 ± 0.21

NeuroHarmonize	58.57 ± 11.56	73.04 ± 14.24	64.25 ± 13.33	51.05 ± 11.93	-1.01 ± 0.18	-1.16 ± 0.19	-1.08 ± 0.18	-1.04 ± 0.21
OPN ComBat	58.16 ± 12.12	72.71 ± 15.02	64.96 ± 13.98	52.24 ± 12.42	-1.02 ± 0.19	-1.16 ± 0.2	-1.07 ± 0.19	-1.04 ± 0.21
AutoComBat	58.29 ± 11.42	72.97 ± 14.21	65.37 ± 13.23	52.24 ± 11.72	-1.01 ± 0.18	-1.16 ± 0.19	-1.07 ± 0.18	-1.04 ± 0.2
RELIEF	61.14 ± 13.21	75.53 ± 16.1	68.08 ± 14.95	54.21 ± 13.69	-1.04 ± 0.15	-1.17 ± 0.16	-1.08 ± 0.13	-1.07 ± 0.19

Supplementary Table 3: Regression results for the associations of age with CBF and log-transformed sCoV per harmonisation method, including interaction effects compared to unharmonised data. *ACA: anterior cerebral artery; CBF: cerebral blood flow; CI: confidence interval; GM: grey matter; MCA: middle cerebral artery; PCA: posterior cerebral artery; sCoV: spatial coefficient of variation.*

CBF (mL/100g/min)	Method	Beta	CI	R^2	P-value	Interaction (P-value)
GM	Unharmonised	-0.37	-0.41, -0.34	0.13	< 0.001	-
	NeuroComBat	-0.45	-0.49, -0.41	0.15	< 0.001	0.002
	CovBat	-0.45	-0.49, -0.41	0.15	< 0.001	0.002
	NeuroHarmonize	-0.43	-0.47, -0.39	0.13	< 0.001	0.02
	OPNested ComBat	-0.42	-0.46, -0.38	0.12	< 0.001	0.05
	AutoComBat	-0.47	-0.51, -0.44	0.17	< 0.001	<0.001
	RELIEF	-0.28	-0.33, -0.23	0.04	< 0.001	0.003
ACA	Unharmonised	-0.31	-0.34, -0.28	0.13	< 0.001	-
	NeuroComBat	-0.30	-0.34, -0.27	0.09	< 0.001	0.69
	CovBat	-0.30	-0.34, -0.27	0.09	< 0.001	0.69
	NeuroHarmonize	-0.30	-0.33, -0.26	0.09	< 0.001	0.49
	OPNested ComBat	-0.28	-0.32, -0.25	0.08	< 0.001	0.25
	AutoComBat	-0.32	-0.36, -0.29	0.11	< 0.001	0.69
	RELIEF	-0.17	-0.21, -0.13	0.02	< 0.001	< 0.001
MCA	Unharmonised	-0.31	-0.34, -0.28	0.12	< 0.001	-
	NeuroComBat	-0.39	-0.42, -0.35	0.13	< 0.001	0.001

	CovBat	-0.39	-0.42, -0.35	0.13	< 0.001	0.001
	NeuroHarmonize	-0.38	-0.41, -0.34	0.13	< 0.001	0.003
	OPNested ComBat	-0.37	-0.41, -0.33	0.12	< 0.001	0.01
	AutoComBat	-0.41	-0.45, -0.38	0.16	< 0.001	< 0.001
	RELIEF	-0.24	-0.29, -0.2	0.04	< 0.001	0.002
PCA	Unharmonised	-0.29	-0.33, -0.25	0.07	< 0.001	-
	NeuroComBat	-0.34	-0.39, -0.3	0.09	< 0.001	0.046
	CovBat	-0.34	-0.38, -0.3	0.09	< 0.001	0.048
	NeuroHarmonize	-0.31	-0.35, -0.27	0.07	< 0.001	0.44
	OPNested ComBat	-0.29	-0.33, -0.25	0.06	< 0.001	0.94
	AutoComBat	-0.35	-0.39, -0.31	0.09	< 0.001	0.037
	RELIEF	-0.19	-0.24, -0.14	0.02	< 0.001	0.002

**sCoV (log(σ/μ))**	**Method**	**Beta**	**CI**	**R^2**	**P-value**	
GM	Unharmonised	20.53	19.44, 21.61	0.32	< 0.001	-
	NeuroComBat	14.00	11.16, 16.84	0.03	< 0.001	< 0.001
	CovBat	13.97	11.14, 16.81	0.03	< 0.001	< 0.001
	NeuroHarmonize	10.77	7.89, 13.65	0.02	< 0.001	< 0.001
	OPNested ComBat	11.54	8.6, 14.48	0.02	< 0.001	< 0.001
	AutoComBat	15.96	13.17, 18.76	0.04	< 0.001	0.003
	RELIEF	13.28	8.78, 17.77	0.01	< 0.001	0.002
ACA	Unharmonised	18.75	17.75, 19.74	0.32	< 0.001	-
	NeuroComBat	8.74	6.03, 11.46	0.01	< 0.001	< 0.001
	CovBat	8.72	6.01, 11.43	0.01	< 0.001	< 0.001
	NeuroHarmonize	6.92	4.19, 9.65	0.01	< 0.001	< 0.001
	OPNested ComBat	6.62	3.81, 9.42	0.01	< 0.001	< 0.001
	AutoComBat	8.36	5.66, 11.05	0.01	< 0.001	< 0.001
	RELIEF	5.41	1.19, 9.63	0.00	0.012	< 0.001
MCA	Unharmonised	18.91	17.84, 19.98	0.29	< 0.001	-
	NeuroComBat	4.43	1.52, 7.34	0.00	0.003	< 0.001

	CovBat	4.41	1.5, 7.31	0.00	0.003	< 0.001
	NeuroHarmonize	2.08	-0.85, 5.01	0.00	0.163	< 0.001
	OPNested ComBat	2.96	-0.05, 5.98	0.00	0.054	< 0.001
	AutoComBat	4.58	1.68, 7.48	0.00	0.002	< 0.001
	RELIEF	5.73	0.28, 11.19	0.00	0.040	< 0.001
PCA	Unharmonised	18.02	17.06, 18.98	0.32	< 0.001	-
	NeuroComBat	14.07	11.66, 16.49	0.04	< 0.001	0.003
	CovBat	14.05	11.64, 16.46	0.04	< 0.001	0.003
	NeuroHarmonize	10.95	8.48, 13.43	0.03	< 0.001	< 0.001
	OPNested ComBat	13.87	11.39, 16.35	0.04	< 0.001	0.002
	AutoComBat	18.85	16.5, 21.2	0.08	< 0.001	0.53
	RELIEF	13.33	9.74, 16.92	0.02	< 0.001	0.11

Supplementary Table 4: BAG and MAE differences between harmonisation methods in the testing dataset, for ASL-only (A) and T1w+FLAIR+ASL features (B). *ASL: arterial spin labelling; BAG: Brain age gap; CI: confidence interval; FLAIR: Fluid attenuated inversion recovery; MAE: mean absolute error; T1w: T1-weighted.*

ASL-only	BAG difference (mean [CI])	P-value	MAE difference (mean [CI])	P-value
NeuroComBat - Unharmonised	-2.13 [-3.02, -1.23]	< 0.001	-4.69 [-5.24, -4.15]	< 0.001
CovBat - Unharmonised	-2.48 [-3.37, -1.59]	< 0.001	-4.66 [-5.2, -4.12]	< 0.001
NeuroHarmonize - Unharmonised	-2.37 [-3.26, -1.48]	< 0.001	-4.76 [-5.31, -4.22]	< 0.001
OPNested ComBat - Unharmonised	-2.55 [-3.44, -1.65]	< 0.001	-4.54 [-5.08, -4]	< 0.001
AutoComBat - Unharmonised	-1.63 [-2.52, -0.74]	< 0.001	-4.77 [-5.31, -4.23]	< 0.001
RELIEF - Unharmonised	-5.72 [-6.62, -4.83]	< 0.001	-2.3 [-2.84, -1.76]	< 0.001
CovBat - NeuroComBat	-0.35 [-1.24, 0.54]	0.909	0.04 [-0.51, 0.58]	1.000
NeuroHarmonize - NeuroComBat	-0.24 [-1.13, 0.65]	0.985	-0.07 [-0.61, 0.47]	1.000
OPNested ComBat - NeuroComBat	-0.42 [-1.31, 0.47]	0.812	0.15 [-0.39, 0.7]	0.981
AutoComBat - NeuroComBat	0.5 [-0.4, 1.39]	0.655	-0.08 [-0.62, 0.47]	1.000
RELIEF - NeuroComBat	-3.59 [-4.49, -2.7]	< 0.001	2.39 [1.85, 2.94]	< 0.001
NeuroHarmonize - CovBat	0.11 [-0.78, 1]	1.000	-0.11 [-0.65, 0.44]	0.998
OPNested ComBat - CovBat	-0.07 [-0.96, 0.83]	1.000	0.12 [-0.43, 0.66]	0.995
AutoComBat - CovBat	0.85 [-0.04, 1.74]	0.076	-0.11 [-0.66, 0.43]	0.997
RELIEF - CovBat	-3.24 [-4.14, -2.35]	< 0.001	2.36 [1.82, 2.9]	< 0.001
OPNested ComBat - NeuroHarmonize	-0.18 [-1.07, 0.72]	0.997	0.22 [-0.32, 0.77]	0.889
AutoComBat - NeuroHarmonize	0.74 [-0.16, 1.63]	0.183	-0.01 [-0.55, 0.54]	1.000

RELIEF - NeuroHarmonize	-3.35 [-4.25, -2.46]	< 0.001	2.46 [1.92, 3.01]	< 0.001
AutoComBat - OPNested ComBat	0.92 [0.02, 1.81]	0.040	-0.23 [-0.77, 0.31]	0.875
RELIEF - OPNested ComBat	-3.18 [-4.07, -2.28]	< 0.001	2.24 [1.7, 2.78]	< 0.001
RELIEF - AutoComBat	-4.09 [-4.98, -3.2]	< 0.001	2.47 [1.93, 3.01]	< 0.001
**T1w+FLAIR+ASL**	**BAG difference (mean [CI])**	**P-value**	**MAE difference (mean [CI])**	**P-value**
NeuroComBat - Unharmonised	-0.12 [-0.87, 0.63]	1.000	0.3 [-0.19, 0.79]	0.570
NeuroComBat (all features) - Unharmonised	5.19 [4.44, 5.94]	< 0.001	-0.57 [-1.05, -0.08]	0.009
CovBat - Unharmonised	0.34 [-0.42, 1.09]	0.880	0.16 [-0.33, 0.65]	0.975
NeuroHarmonize - Unharmonised	-0.06 [-0.82, 0.69]	1.000	0.34 [-0.15, 0.82]	0.418
OPNested ComBat - Unharmonised	0.14 [-0.62, 0.89]	0.999	0.27 [-0.22, 0.75]	0.711
AutoComBat - Unharmonised	0.27 [-0.48, 1.03]	0.957	0.16 [-0.33, 0.64]	0.976
RELIEF - Unharmonised	-0.37 [-1.12, 0.39]	0.822	0.54 [0.06, 1.03]	0.017
NeuroComBat (all features) - NeuroComBat	5.31 [4.56, 6.06]	< 0.001	-0.87 [-1.35, -0.38]	< 0.001
CovBat - NeuroComBat	0.46 [-0.3, 1.21]	0.599	-0.14 [-0.63, 0.35]	0.988
NeuroHarmonize - NeuroComBat	0.06 [-0.7, 0.81]	1.000	0.04 [-0.45, 0.52]	1.000
OPNested ComBat - NeuroComBat	0.26 [-0.5, 1.01]	0.970	-0.03 [-0.52, 0.45]	1.000
AutoComBat - NeuroComBat	0.39 [-0.36, 1.15]	0.762	-0.14 [-0.63, 0.34]	0.988
RELIEF - NeuroComBat	-0.25 [-1, 0.51]	0.976	0.24 [-0.24, 0.73]	0.803
CovBat - NeuroComBat (all features)	-4.86 [-5.61, -4.1]	< 0.001	0.73 [0.24, 1.21]	< 0.001
NeuroHarmonize - NeuroComBatAllFeatures	-5.25 [-6.01, -4.5]	< 0.001	0.9 [0.42, 1.39]	< 0.001
OPNested ComBat - NeuroComBat (all features)	-5.05 [-5.81, -4.3]	< 0.001	0.83 [0.35, 1.32]	< 0.001
AutoComBat - NeuroComBat (all features)	-4.92 [-5.67, -4.16]	< 0.001	0.73 [0.24, 1.21]	< 0.001
RELIEF- NeuroComBat (all features)	-5.56 [-6.31, -4.8]	< 0.001	1.11 [0.62, 1.6]	< 0.001
NeuroHarmonize - CovBat	-0.4 [-1.15, 0.35]	0.748	0.18 [-0.31, 0.66]	0.958
OPNested ComBat - CovBat	-0.2 [-0.95, 0.55]	0.993	0.11 [-0.38, 0.59]	0.998
AutoComBat - CovBat	-0.06 [-0.82, 0.69]	1.000	0 .01[-0.49, 0.48]	1.000
RELIEF - CovBat	-0.7 [-1.46, 0.05]	0.090	0.38 [-0.1, 0.87]	0.251
OPNested ComBat - NeuroHarmonize	0.2 [-0.55, 0.95]	0.993	-0.07 [-0.55, 0.42]	1.000
AutoComBat - NeuroHarmonize	0.34 [-0.42, 1.09]	0.877	-0.18 [-0.66, 0.31]	0.956
RELIEF - NeuroHarmonize	-0.3 [-1.06, 0.45]	0.927	0.21 [-0.28, 0.69]	0.905
AutoComBat - OPNested ComBat	0.14 [-0.62, 0.89]	0.999	-0.11 [-0.59, 0.38]	0.998
RELIEF - OPNested ComBat	-0.5 [-1.26, 0.25]	0.468	0.27 [-0.21, 0.76]	0.677
RELIEF - AutoComBat	-0.64 [-1.39, 0.11]	0.166	0.38 [-0.1, 0.87]	0.246

Supplementary Table 5: BAG and MAE of the validation and testing cohorts separately per harmonisation method, obtained using ASL-only (A) features or T1w+FLAIR+ASL (B) features to predict brain age. *ASL: arterial spin labelling; BAG: Brain age gap; FLAIR: Fluid attenuated inversion recovery; MAE: mean absolute error; T1w: T1-weighted.*

A. Cohort	Method	BAG (μ ± σ)	MAE (μ ± σ)
Validation	Unharmonised	-0.23 ± 13.03	10.65 ± 7.5
	NeuroComBat	-0.43 ± 13.88	11.32 ± 8.04
	CovBat	-0.22 ± 13.74	11.19 ± 7.96
	NeuroHarmonize	-0.24 ± 13.90	11.37 ± 8.00
	OPNested ComBat	-0.12 ± 13.62	11.15 ± 7.81
	AutoComBat	-0.18 ± 13.06	10.62 ± 7.61
	RELIEF	-0.22 ± 12.91	10.42 ± 7.62
HELIUS	Unharmonised	1.86 ± 10.89	9.24 ± 6.04

	NeuroComBat	-0.89 ± 8.23	6.68 ± 4.88
	CovBat	-1.04 ± 8.14	6.59 ± 4.88
	NeuroHarmonize	-0.33 ± 8.01	6.54 ± 4.63
	OPNested ComBat	-0.66 ± 8.32	6.64 ± 5.05
	AutoComBat	-0.29 ± 8.35	6.67 ± 5.02
	RELIEF	-2.22 ± 8.82	7.4 ± 5.27
SABRE	Unharmonised	8.91 ± 6.06	9.73 ± 4.65
	NeuroComBat	-2.36 ± 7.23	6.07 ± 4.58
	CovBat	-2.75 ± 7.18	6.15 ± 4.62
	NeuroHarmonize	-2.85 ± 6.98	5.99 ± 4.57
	OPNested ComBat	-2.91 ± 7.31	6.16 ± 4.89
	AutoComBat	-1.46 ± 7.15	5.73 ± 4.51
	RELIEF	-8.98 ± 7.51	9.93 ± 6.19
EDIS	Unharmonised	-13.72 ± 13.41	16.3 ± 10.10
	NeuroComBat	-1.85 ± 8.03	6.53 ± 5.02
	CovBat	-2.4 ± 7.96	6.65 ± 4.98
	NeuroHarmonize	-2.53 ± 7.73	6.44 ± 4.95
	OPNested ComBat	-2.82 ± 8.26	6.91 ± 5.32
	AutoComBat	-1.61 ± 8.21	6.64 ± 5.07
	RELIEF	-4.1 ± 10.33	8.78 ± 6.79
Insight 46	Unharmonised	-3.09 ± 13.7	11.25 ± 8.38
	NeuroComBat	-0.4 ± 8.30	6.39 ± 5.30
	CovBat	-0.8 ± 8.30	6.48 ± 5.23
	NeuroHarmonize	-1.04 ± 8.33	6.51 ± 5.28
	OPNested ComBat	-1.07 ± 8.74	6.78 ± 5.59
	AutoComBat	-0.71 ± 8.29	6.56 ± 5.11
	RELIEF	-3.05 ± 10.34	8.78 ± 6.24

B. Cohort	Method	BAG (μ ± σ)	MAE (μ ± σ)
Validation	Unharmonised	-0.07 ± 6.47	5.11 ± 3.98
	NeuroComBat	0.11 ± 6.69	5.33 ± 4.03
	CovBat	-0.08 ± 6.52	5.13 ± 4.01
	NeuroHarmonize	0.11 ± 6.78	5.39 ± 4.11
	OPNested ComBat	-0.06 ± 6.56	5.15 ± 4.06
	AutoComBat	-0.05 ± 6.5	5.12 ± 4
	RELIEF	-0.05 ± 6.61	5.21 ± 4.06
	NeuroComBat (all features)	0.00 ± 7.3	5.77 ± 4.47
HELIUS	Unharmonised	2.56 ± 6.8	5.76 ± 4.43
	NeuroComBat	1.84 ± 7.2	5.83 ± 4.61
	CovBat	2.25 ± 7.16	5.93 ± 4.59
	NeuroHarmonize	1.85 ± 7.19	5.89 ± 4.52
	OPNested ComBat	2.24 ± 7.12	5.9 ± 4.56
	AutoComBat	2.23 ± 7.04	5.82 ± 4.54
	RELIEF	1.86 ± 7.2	5.88 ± 4.55
	NeuroComBat (all features)	4.04 ± 7.18	6.74 ± 4.72
SABRE	Unharmonised	-2.98 ± 6.35	5.42 ± 4.44
	NeuroComBat	-3.53 ± 7.13	6.02 ± 5.2
	CovBat	-3.04 ± 6.99	5.72 ± 5.03
	NeuroHarmonize	-3.46 ± 7.12	6.01 ± 5.14
	OPNested ComBat	-3.29 ± 7.02	5.86 ± 5.08

	AutoComBat	-3.11 ± 6.95	5.74 ± 5.01
	RELIEF	-3.75 ± 7.33	6.21 ± 5.4
	NeuroComBat (all features)	3.06 ± 4.66	4.52 ± 3.27
EDIS	Unharmonised	-1.25 ± 7.19	5.62 ± 4.64
	NeuroComBat	0.16 ± 7.52	5.92 ± 4.63
	CovBat	0.6 ± 7.53	5.92 ± 4.68
	NeuroHarmonize	0.31 ± 7.55	5.96 ± 4.63
	OPNested ComBat	0.29 ± 7.59	5.99 ± 4.66
	AutoComBat	0.52 ± 7.55	5.95 ± 4.66
	RELIEF	-0.16 ± 7.79	6.15 ± 4.77
	NeuroComBat (all features)	3.6 ± 5.21	5.21 ± 3.6
Insight46	Unharmonised	-6.94 ± 5.61	7.46 ± 4.89
	NeuroComBat	-6.82 ± 6.26	7.51 ± 5.41
	CovBat	-6.35 ± 6.23	7.1 ± 5.35
	NeuroHarmonize	-6.84 ± 6.51	7.6 ± 5.6
	OPNested ComBat	-6.67 ± 6.55	7.46 ± 5.62
	AutoComBat	-6.44 ± 6.24	7.24 ± 5.29
	RELIEF	-7.55 ± 6.68	8.24 ± 5.82
	NeuroComBat (all features)	3.72 ± 3.69	4.55 ± 2.6

Supplementary Table 6: Uncorrected BAG and MAE per harmonisation method for the testing sets, obtained using ASL-only or T1w+FLAIR+ASL features to predict brain age. Additional results of harmonisation of all features using NeuroComBat have been included under ‘NeuroComBat (all features)’. Note that the validation set was not corrected for age-bias. *ASL: arterial spin labelling; BAG: brain-predicted age gap; FLAIR: fluid attenuated inversion recovery; MAE: mean absolute error; T1w: T1-weighted.*

**ASL-only**	**BAG (μ ± σ)**	**MAE (μ ± σ)**	R^2
Unharmonised	-10.46 ± 14.72	13.24 ± 12.28	0.02
NeuroComBat	-13.73 ± 10.21	14.78 ± 8.62	0.05
CovBat	-13.82 ± 10.19	14.86 ± 8.61	0.05
NeuroHarmonize	-13.92 ± 10.3	15.08 ± 8.52	0.03
OPNested ComBat	-13.44 ± 10.46	14.66 ± 8.68	0.04
AutoComBat	-12.02 ± 9.96	13.31 ± 8.17	0.08
RELIEF	-16.01 ± 11.63	17.01 ± 10.12	0.02
**T1w+FLAIR+ASL**	**BAG (μ ± σ)**	**MAE (μ ± σ)**	R^2
Unharmonised	-4.70 ± 7.88	7.40 ± 5.42	0.30
NeuroComBat	-4.70 ± 8.21	7.49 ± 5.78	0.31

CovBat	-4.38 ± 8.14	7.28 ± 5.70	0.32
NeuroHarmonize	-4.71 ± 8.24	7.51 ± 5.81	0.31
OPNested ComBat	-4.50 ± 8.23	7.37 ± 5.80	0.31
AutoComBat	-4.45 ± 8.13	7.31 ± 5.70	0.32
RELIEF	-5.01 ± 8.45	7.74 ± 6.05	0.30
NeuroComBat (all features)	-0.18 ± 6.20	4.71 ± 4.02	0.49
